# Deep learning techniques for extreme rainfall prediction: a review of state-of-the-art

**DOI:** 10.3389/frai.2026.1908848

**Published:** 2026-08-27

**Authors:** Braiton U. Mukhalela, Serestina Viriri, David Ndzi, Michael Gebreslasie, Muhammad Zeeshan Shakir, Naeem Ramzan, Fahad Ayaz, Mary Lynch, Llinos Haf Spencer, Saloshni Naidoo, Nisha Nadesan-Reddy, Ozayr Mahomed, Natalie Dickinson, Fiona Henriquez

**Affiliations:** 1 Discipline of Computer Science, School of Agriculture and Science, University of KwaZulu-Natal, Durban, South Africa; 2 School of Electrical and Mechanical Engineering, University of Portsmouth, England, United Kingdom; 3 Discipline of Geography, School of Agriculture and Science, University of KwaZulu-Natal, Durban, South Africa; 4 School of Computing, Engineering and Physical Sciences, University of the West of Scotland, Paisley, United Kingdom; 5 Royal College of Surgeons in Ireland, University of Medicine and Health Sciences, Dublin, Ireland; 6 Discipline of Public Health, Institute for Global Health, University of KwaZulu-Natal, Durban, South Africa; 7 School of Health and Life Sciences, University of the West of Scotland, Paisley, United Kingdom; 8 Civil and Environmental Engineering Department, University of Strathclyde, Glasgow, United Kingdom

**Keywords:** artificial intelligence, deep learning, early warning systems, extreme rainfall prediction, multimodal data fusion, numerical weather prediction, operational forecasting, physics-informed learning

## Abstract

Extreme rainfall events (ERES) are among the most challenging hydro-meteorological phenomena to forecast because the complex, nonlinear atmospheric processes involved span multiple spatial and temporal scales and are further intensified by rising climate variability. While Numerical Weather Prediction (NWP) models offer physically consistent representations of atmospheric dynamics, they still struggle to resolve localized convection, rapidly evolving storm systems, and rare, high-intensity precipitation events. This study systematically reviews recent advances in Artificial Intelligence (AI) for extreme rainfall prediction published between 2020 and 2026, using a transparent literature search strategy, predefined screening criteria, quality assessment, and a structured literature review matrix. The review synthesizes evidence from a final evidence base of 139 studies across several dimensions, including deep learning (DL) methodologies, multimodal data fusion, forecasting horizons, operational deployment, uncertainty quantification, and emerging intelligent forecasting paradigms. The analysis reveals a transition from deterministic DI models to hybrid, physics-informed, uncertainty-aware, and operational forecasting systems that increasingly integrate AI with physical knowledge and heterogeneous environmental observations. Despite notable progress, persistent challenges remain, including data scarcity, class imbalance, model generalization, physical consistency, uncertainty estimation, transferability, and the lack of standardized evaluation frameworks. This review provides a unified synthesis of current methodologies. It identifies key research gaps, highlighting the need for evaluation frameworks that address physical plausibility, uncertainty quantification, reliability, transferability, and operational relevance. The findings indicate that future extreme rainfall prediction systems should advance beyond accuracy-focused evaluation toward integrated, trustworthy, uncertainty-aware, and physically consistent forecasting approaches.

## 1 Introduction

Extreme rainfall events (EREs) have attracted significant scientific and societal attention because of their rising frequency, greater spatial variability, and substantial socio-economic impacts. Recent flash floods in Mediterranean Europe, intensified monsoon rainfall in Asia, and severe flood disasters in southern Africa collectively illustrate the escalating hydro-climatic risks associated with global warming (Mastrantonas et al., 2021). Although thermodynamic intensification from increased atmospheric moisture is widely recognized as a primary driver of heavier precipitation, localized extreme rainfall is also shaped by complex interactions among atmospheric dynamics, moisture transport, topography, and land-surface processes. These interactions remain only partially understood and continue to challenge both scientific research and operational forecasting. Attribution studies linking the 2022 KwaZulu-Natal floods to climate change (Pinto et al., 2022), together with evidence of evolving rainfall non-stationarity along South Africa's eastern coastline (Johnson et al., 2025), underscore the growing need for forecasting systems that can represent both changing climatic conditions and local hydro-meteorological processes. Globally, similar research indicates that flood impacts increasingly result from the combined effects of rainfall intensity, exposure, infrastructure vulnerability, and adaptive capacity, rather than from rainfall magnitude alone (Byaruhanga et al., 2025; Yu H. et al., 2025).

Forecasting extreme rainfall remains one of the most complex challenges in operational meteorology. Numerical Weather Prediction (NWP) models provide physically based representations of atmospheric processes. Yet, they continue to face difficulties in accurately resolving highly localized convective initiation, rapid storm evolution, and the nonlinear interactions underlying rare, high-impact precipitation extremes. These limitations are especially pronounced during the nowcasting period (0–6 h), when small uncertainties in moisture initialization, boundary-layer evolution, or convective triggering can lead to substantial errors in rainfall timing, location, and intensity. Although significant progress has been made in synoptic-scale forecasting (Bauer et al., 2015), persistent challenges related to convective parameterization and grid-scale smoothing remain well documented (Doswell, 2001; Wilson et al., 1998; Golding, 1998; Satyanarayana and Kar, 2016; Brotzge et al., 2023). Consequently, reliably predicting low-probability but high-impact rainfall events remains a major scientific and operational challenge (Verma et al., 2023).

The rapid advancement of artificial intelligence (AI), particularly deep learning (DL), has substantially transformed precipitation forecasting. Deep learning enables models to capture complex nonlinear relationships directly from diverse environmental observations, including weather radar, satellite imagery, reanalysis products, and *in-situ* sensor networks. Early recurrent architectures, such as Long Short-Term Memory (LSTM) networks, demonstrated strong capabilities for modeling temporal rainfall evolution (Frame et al., 2021), while convolutional neural networks (CNNs) improved spatial feature extraction from gridded meteorological data. More recently, transformer architectures have introduced attention mechanisms that model long-range spatio-temporal dependencies. Generative adversarial networks (GANs) and diffusion models have further enhanced the representation of rare, high-intensity precipitation events by generating more realistic rainfall distributions (Zheng et al., 2025; Fang et al., 2025). Recent research suggests that future forecasting systems will increasingly use foundation models trained on large-scale Earth observation archives, together with adaptive Mixture-of-Experts (MoE) architectures that dynamically route meteorological scenarios to specialized expert networks (Chakraborty et al., 2025; Xiao et al., 2026; Jiang et al., 2025; Gan et al., 2025). Collectively, these developments indicate that progress is being made by integrating complementary modeling paradigms, rather than by replacing earlier architectures, to represent increasingly complex atmospheric processes.

Despite notable methodological advances, the literature remains fragmented across architectural families, forecasting horizons, climatic regions, observational datasets, and operational objectives. This fragmentation complicates the comparison of competing approaches and hinders the identification of broader scientific trends that support reliable extreme rainfall prediction (ERP). Persistent challenges include severe class imbalance associated with rare extreme events, limited model transferability across hydro-climatic regions, inadequate incorporation of physical constraints, inconsistent benchmarking protocols, insufficient attention to forecast uncertainty, limited interpretability, and limited operational usability. As AI becomes increasingly central to early warning systems (EWS) and disaster risk reduction, forecasting performance alone is insufficient. Operational forecasting systems must also be trustworthy, physically consistent, uncertainty-aware, and capable of supporting transparent decision-making.

These challenges underscore the need for a comprehensive synthesis of recent advances in AI-based ERP. Rather than reviewing individual model families in isolation, this study integrates developments published between 2020 and 2026 across multiple complementary dimensions, including DL architectures, forecasting horizons, climatic settings, multimodal environmental observations, operational application domains, evaluation strategies, methodological innovations, and future research directions. Particular emphasis is placed on the ongoing transition from deterministic DL models to hybrid, physics-informed, uncertainty-aware, and operationally deployable forecasting systems that support real-world hydro-meteorological decision-making.

This review uses a structured literature review framework. The final evidence base comprises 139 peer-reviewed studies drawn from major scientific databases, including IEEE Xplore, ScienceDirect, Scopus, Web of Science, Google Scholar, and the American Meteorological Society Digital Library. The studies are systematically evaluated based on their modeling approaches, environmental data sources, treatment of extreme rainfall, evaluation methodologies, and operational relevance. Rather than seeking a universally superior forecasting architecture, the review identifies broader scientific trends shaping the evolution of AI for ERP. These trends include multimodal data fusion, physics-informed learning, trustworthy AI, adaptive forecasting systems, and greater integration with operational NWP.

The remainder of this review is organized as follows. Sections 2, 3 present the review methodology and the physical foundations of ERP. Sections 4 through 8 synthesize recent advances in DL architectures, forecasting horizons, climatic settings, methodological innovations, and operational application domains. Sections 9, 10 critically examine the principal scientific challenges, emerging research directions, and future priorities for trustworthy and operational AI in ERP. Section 11 concludes by summarizing the major findings and outlining future opportunities to develop robust, transferable, and operationally deployable forecasting systems.

## 2 Systematic review design and methodology

### 2.1 Review design and scope

This study adopts a structured systematic literature review with thematic synthesis to examine recent advances in AI and DL for ERP. Unlike conventional narrative reviews, the proposed design systematically organizes the literature into coherent thematic dimensions, enabling comparative analysis of methodological developments, application domains, operational considerations, and emerging research trends. The review focuses on publications from 2020 to 2026, reflecting the rapid evolution of AI-driven rainfall forecasting during this period. Particular emphasis is placed on recent advances that have transformed the field from conventional DL models toward more integrated, physically consistent, and operationally deployable forecasting systems. The overall review design, scope, and principal synthesis dimensions adopted in this study are summarized in Table 1.

**Table 1:** Review framework and scope adopted in this study.

Component	Description
Review type	Structured systematic literature review with thematic synthesis
Publication period	2020–2026
Primary domain	AI and DL for extreme rainfall prediction
Principal objectives	Analyze advances in AI architectures, forecasting horizons, climatic applications, operational deployment, and future research directions
Main synthesis dimensions	Architecture, forecasting horizon, climate context, application domain, evaluation strategy, operational relevance, and research priorities
Key emphasis	Representation of high-intensity rainfall extremes, uncertainty-aware forecasting, multimodal data fusion, and operational decision support
Overall motivation	Identify scientific trends, methodological gaps, and future opportunities for developing trustworthy and operational AI-based rainfall forecasting systems

### 2.2 Literature sources and search strategy

A comprehensive literature search was conducted to identify studies on AI applications for ERP. The search followed a transparent, reproducible strategy to maximize coverage and uphold scientific rigor. Publications were sourced from major bibliographic databases, with additional records identified through reference chaining and selected preprint repositories to capture emerging methodological developments. The search used keywords related to extreme rainfall, forecasting applications, AI methodologies, and environmental data sources. These terms were chosen to encompass advances in DL, probabilistic forecasting, multimodal data fusion, and operational weather prediction published between January 2020 and June 2026. All retrieved records were consolidated, duplicates removed, and further relevant studies identified through forward and backward citation tracking. Table 2 summarizes the literature sources, keyword categories, and publication period adopted in this review.

**Table 2:** Literature sources and search strategy adopted in this review.

Search element	Description
Primary databases	IEEE Xplore, ScienceDirect, Web of Science, Scopus, Google Scholar, and AMS Digital Library
Supplementary sources	SpringerLink, arXiv (methodologically significant studies), forward and backward citation tracking
Extreme rainfall terms	“extreme rainfall,” “heavy rainfall,” “precipitation extremes,” “extreme precipitation,” “flash floods”
Forecasting terms	“prediction,” “nowcasting,” “early warning systems,” “precipitation forecasting,” “hydrological forecasting,” “radar echo extrapolation”
Artificial intelligence terms	“CNN,” “LSTM,” “ConvLSTM,” “Transformer,” “GAN,” “diffusion model,” “foundation model,” “Mixture-of-Experts,” “physics-informed learning,” “neural operator,” “trustworthy AI,” “explainable AI,” ‘uncertainty quantification,” “evidential learning”
Environmental data terms	Radar, satellite observations, reanalysis, GNSS-PWV, NWP, weather stations, terrain, and multimodal data fusion
Publication window	January 2020 - June 2026

### 2.3 Inclusion and exclusion criteria

Explicit inclusion and exclusion criteria were established before the literature screening to ensure methodological consistency, reduce selection bias, and align with the objectives of this review. These criteria helped identify studies that demonstrated scientific rigor, direct relevance to AI-based ERP, and suitability for comparative thematic synthesis. Studies outside the review scope or failing to satisfy the predefined inclusion and eligibility criteria were excluded. During the full-text eligibility assessment, studies were excluded if they fell outside the scope of ERP, employed forecasting approaches unrelated to AI or DL, provided insufficient methodological detail for meaningful comparison, were duplicate publications, had unavailable full texts, or failed to satisfy the predefined inclusion and eligibility criteria. The complete screening criteria adopted during the study selection process are summarized in Table 3.

**Table 3:** Study inclusion and exclusion criteria adopted in this review.

Category	Criterion	Purpose
Inclusion	Focus on extreme rainfall or related hydro-meteorological hazards	Ensures thematic relevance
	Uses deep learning or contemporary AI methods (e.g., hybrid AI, AI–NWP integration, physics-informed learning)	Matches the review scope
	Reports quantitative evaluation results	Supports comparative analysis
	Provides sufficient methodological detail	Enables reproducibility and thematic synthesis
Exclusion	General weather forecasting without an extreme rainfall focus	Avoids scope drift
	Conventional statistical or physics-based methods only	Outside the review scope
	Insufficient methodological transparency or validation	Ensures methodological quality
	Editorials, abstracts, and non-peer-reviewed publications (except influential methodological preprints)	Ensures scientific reliability

### 2.4 Study selection and screening process

A PRISMA-inspired workflow was implemented to improve the transparency and reproducibility of the literature screening process. Records from multiple databases and supplementary sources were consolidated, duplicates were removed, and the remaining studies were screened by title and abstract. Studies deemed eligible then underwent full-text assessment against the predefined inclusion and exclusion criteria outlined in Section 2.3.

Studies that met the selection criteria were analyzed using a structured literature review matrix that documented model architecture, environmental data sources, forecasting horizon, prediction objectives, evaluation metrics, treatment of ERE, operational relevance, uncertainty representation, reported limitations, and key scientific contributions. This framework enabled consistent comparison across diverse AI methodologies and supported the thematic synthesis presented in this review. The literature search results were carefully verified to ensure numerical consistency throughout the PRISMA-inspired workflow. A total of 388 records were initially identified across the selected bibliographic databases. After removing 78 duplicate records, 310 unique studies remained for title and abstract screening. These records then underwent eligibility assessment against the predefined inclusion and exclusion criteria, yielding the final evidence base used in this review.

**Figure 1:**
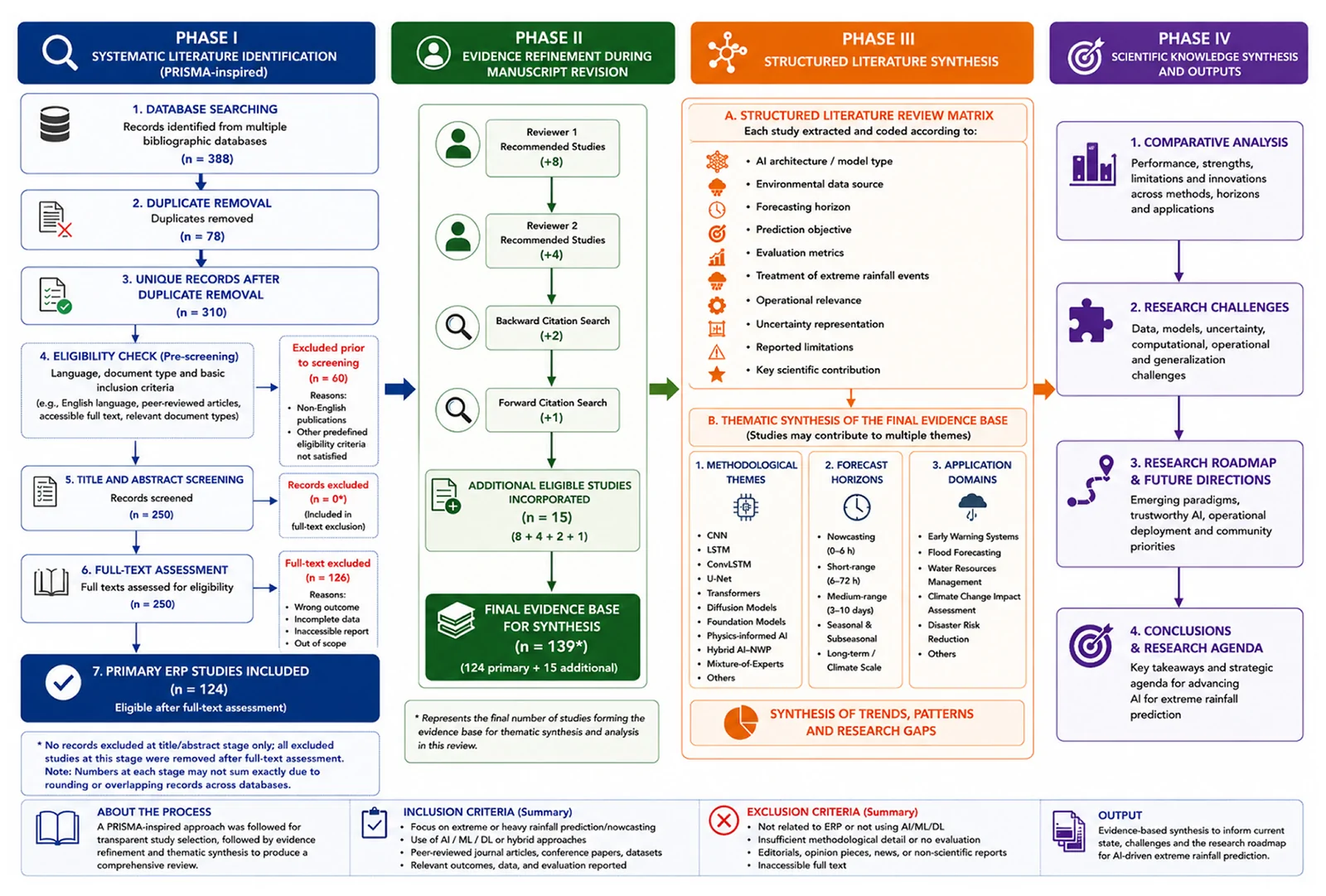
Integrated PRISMA-inspired workflow illustrating literature identification, evidence refinement, structured literature synthesis, and knowledge generation, resulting in a final evidence base of 139 studies used for qualitative synthesis. Figure drawn by authors.

Figure 1 Integrated PRISMA-inspired workflow illustrating the methodology used in this review. Phase I summarizes the systematic identification, screening, and selection of the primary evidence base. Phase II shows the incorporation of additional studies identified through reviewer recommendations and backward/forward citation tracking during manuscript revision. Phase III presents the structured literature review matrix and thematic synthesis that organized the final evidence base into methodological themes, forecasting horizons, and application domains. Phase IV demonstrates how the synthesized evidence informed the comparative analysis, identification of research challenges, development of the research roadmap, and formulation of the study's conclusions. The final evidence base comprised 139 studies, including 124 identified through the systematic search and 15 additional studies incorporated during manuscript revision via citation tracking and reviewer recommendations.

### 2.5 Quality assessment and data extraction

A structured methodological quality assessment was conducted for all eligible studies to support the thematic synthesis and ensure methodological rigor and comprehensive reporting. The assessment considered research objectives, dataset quality, methodological transparency, evaluation methodology, operational relevance, and reproducibility, rather than relying solely on predictive performance, as summarized in Table 4.

**Table 4:** Methodological quality assessment criteria applied during study selection and data extraction.

Assessment criterion	Purpose
Research objective	Verify that the study clearly defines the rainfall prediction problem and forecasting objective.
Dataset quality	Assess the adequacy of environmental datasets, temporal coverage, preprocessing procedures, and data sources.
Methodological transparency	Evaluate whether the artificial intelligence architecture, training strategy, and implementation are described sufficiently for scientific interpretation.
Evaluation methodology	Confirm that recognized forecasting metrics and appropriate validation procedures are reported.
Operational relevance	Assess consideration of uncertainty quantification, physical consistency, interpretability, early warning capability, or operational deployment.
Reproducibility	Evaluate whether sufficient methodological detail is provided to enable independent interpretation or future replication.

The methodological quality assessment supported the critical interpretation, thematic synthesis, and comparative analysis of the included studies. It was not used as an independent criterion for study inclusion or exclusion, and no quantitative quality score or exclusion threshold was applied during the review process. Instead, the assessment provided a qualitative evaluation of methodological strengths, reporting practices, validation procedures, reproducibility, and uncertainty quantification across the included studies. Overall, the quality assessment revealed several recurring methodological limitations in the reviewed literature. Many studies relied on geographically restricted datasets, limiting the generalizability of their findings across climatic regions. External validation using independent datasets was relatively uncommon, and reproducibility was frequently constrained by incomplete reporting of data preprocessing procedures, model hyperparameters, or implementation details. Furthermore, uncertainty quantification and probabilistic verification were inconsistently addressed, and benchmarking against operational forecasting systems remained limited. These observations informed the critical synthesis, the comparative evaluation of DL approaches, and the identification of future research directions presented throughout this review.

Following the quality assessment, data from each study were extracted using a structured literature review matrix that documented model characteristics, environmental data sources, prediction objectives, evaluation metrics, treatment of ERE, uncertainty representation, operational relevance, reported limitations, and key scientific contributions. The extracted information was organized into thematic categories to enable consistent comparison across deterministic, probabilistic, hybrid, and physics-informed forecasting approaches.

## 3 Background on extreme rainfall

### 3.1 Defining extreme rainfall across the literature

The term *extreme rainfall* is prevalent in AI and hydro-meteorological research; however, a universally accepted definition remains absent. Instead, studies define extreme rainfall based on regional climatology, forecasting objectives, data availability, and operational requirements. Consequently, AI models often address distinct prediction problems, complicating direct comparison of reported performance. Table 5 summarizes the principal approaches identified in the reviewed literature and their implications for AI-based forecasting.

**Table 5:** Principal approaches used to define extreme rainfall in the reviewed literature and their implications for AI-based forecasting.

Definition approach	Typical implementation	Representative studies	Implications for AI evaluation
Rainfall intensity thresholds	Predefined rainfall intensity or heavy-rain categories	Lu et al., 2024; Wang Y. V. et al., 2024; Zhang et al., 2025; Fang et al., 2025	Threshold-dependent; may influence CSI, POD, FAR, and FSS. FSS additionally depends on the spatial neighborhood scale adopted during verification, limiting direct cross-study comparison.
Percentile-based climatological thresholds	Percentile indices (e.g., R95p, 95th/99th percentile)	Otero and Horton, 2023; Chandel et al., 2025; Wei and Chiu, 2025; Duncan et al., 2022	Accounts for regional climate variability but depends on dataset-specific thresholds.
Extreme event occurrence	Prediction of event occurrence or frequency	Fan et al., 2024; Xian et al., 2025; Mouassom et al., 2025	Suitable for event detection and early warning but provides limited rainfall magnitude information.
Flood- or impact-oriented definitions	Rainfall–runoff response, flooding, or infrastructure impacts	Frame et al., 2021; Wang et al., 2025; Yousaf et al., 2025	Supports disaster management but depends on hydrological and socioeconomic context.
Operational warning definitions	Warning thresholds or maximum rainfall criteria	Wang et al., 2026; Sahu et al., 2025; De Sousa Araújo et al., 2022	Supports operational forecasting, although warning criteria vary across agencies and regions.

The reviewed studies indicate that the chosen event definition significantly influences model evaluation. Lower rainfall thresholds typically increase the number of positive samples and may influence the Probability of Detection (POD) and False Alarm Ratio (FAR), although the magnitude and direction of these effects depend on the event distribution, dataset characteristics, and model behavior. Conversely, stricter intensity or percentile-based definitions introduce greater class imbalance, making metrics such as the Critical Success Index (CSI) and Fractions Skill Score (FSS) more challenging to optimize. In addition to rainfall thresholds, the Fractions Skill Score (FSS) is also influenced by the spatial neighborhood scale adopted during verification, enabling assessment of forecast skill across multiple spatial resolutions. Probabilistic measures, including the conventional Continuous Ranked Probability Score (CRPS), are likewise affected because they evaluate the entire predictive distribution. Conversely, threshold-weighted or tail-focused probabilistic verification metrics place greater emphasis on accurately forecasting rare, high-impact rainfall events.

Variations in reported forecasting performance often reflect differences in event definition as much as advances in AI methodology. Future benchmarking studies should explicitly report the adopted definition of extreme rainfall, along with the datasets, model architecture, and evaluation metrics, to enhance reproducibility and facilitate fair comparison across forecasting systems.

### 3.2 Physical drivers of extreme rainfall

Extreme rainfall arises from the interaction of thermodynamic and dynamic atmospheric processes operating across multiple spatial and temporal scales. The magnitude and intensity of such events depend on factors such as atmospheric moisture, convective instability, vertical air motion, and large-scale circulation. These factors contribute to substantial variability in rainfall across climatic regions and storm types.

Atmospheric moisture is the primary thermodynamic driver of extreme rainfall. According to the Clausius-Clapeyron relationship, the atmosphere can hold about 6% to 7% more water vapor per degree Celsius of warming, increasing the likelihood of heavier precipitation (Ivancic and Shaw, 2016). However, elevated moisture content alone does not guarantee extreme rainfall; additional favorable atmospheric conditions, such as moisture convergence, upward motion, and convective initiation, are also necessary (Mischell and Soden, 2025; Schoofs et al., 2026).

Large-scale circulation systems, such as monsoons, tropical cyclones, atmospheric rivers, and orographic lifting, regulate moisture transport and storm organization. These systems often produce rainfall intensities that exceed predictions based solely on thermodynamic considerations (Verma et al., 2023; Yu H. et al., 2025; Afandi et al., 2025; Lenderink et al., 2025). The interaction of these processes across scales, together with their sensitivity to initial atmospheric conditions, increases the complexity of ERP.

Therefore, effective forecasting systems must integrate local convective processes with broader atmospheric circulation while preserving the fundamental physical relationships that govern moisture transport and storm development. These requirements underpin many forecasting challenges addressed in the following section and motivate the development of advanced AI methods examined in this study.

### 3.3 Forecasting challenges associated with extreme rainfall

Despite advances in meteorological observations and NWP, forecasting extreme rainfall remains a major challenge in atmospheric science. The rarity, rapid development, and spatial variability of EREs introduce substantial uncertainty, complicating precise predictions of their timing, location, and intensity (Holton, 2013; Doswell, 2001; Otero and Horton, 2023; Harris and Chen, 2025).

Traditional forecasting methods have notable limitations. Numerical Weather Prediction models rely on physical parameterizations that often fail to capture local convective processes. Statistical models, by contrast, assume relationships that may not hold under evolving climatic conditions. Additionally, the infrequency of EREs produces highly imbalanced datasets, complicating model calibration and increasing the risk of missed detections and false alarms. Forecast reliability is further reduced by observational uncertainties, limited monitoring networks, and inconsistencies in spatial and temporal resolution (Bauer et al., 2015; Otero and Horton, 2023; Choi et al., 2025a; Khot et al., 2024; Johnson et al., 2025).

Effective forecasting systems must represent interacting processes across multiple scales, including local convection, mesoscale storm organization, and large-scale atmospheric circulation. These systems must also extract complex spatio-temporal relationships from diverse environmental datasets (Ren et al., 2021; Harris and Chen, 2025; Bentivoglio et al., 2025; Trivedi et al., 2024b). Therefore, advancing ERP depends on models that integrate robust spatio-temporal learning, uncertainty quantification, and adherence to physical principles to enable reliable operational forecasting (Bauer et al., 2015; Bentivoglio et al., 2025; Waqas et al., 2025).

These challenges have accelerated the development of advanced AI-based forecasting methodologies, which are discussed in the following subsection.

### 3.4 Advancements in rainfall prediction methodologies

**Figure 2:**
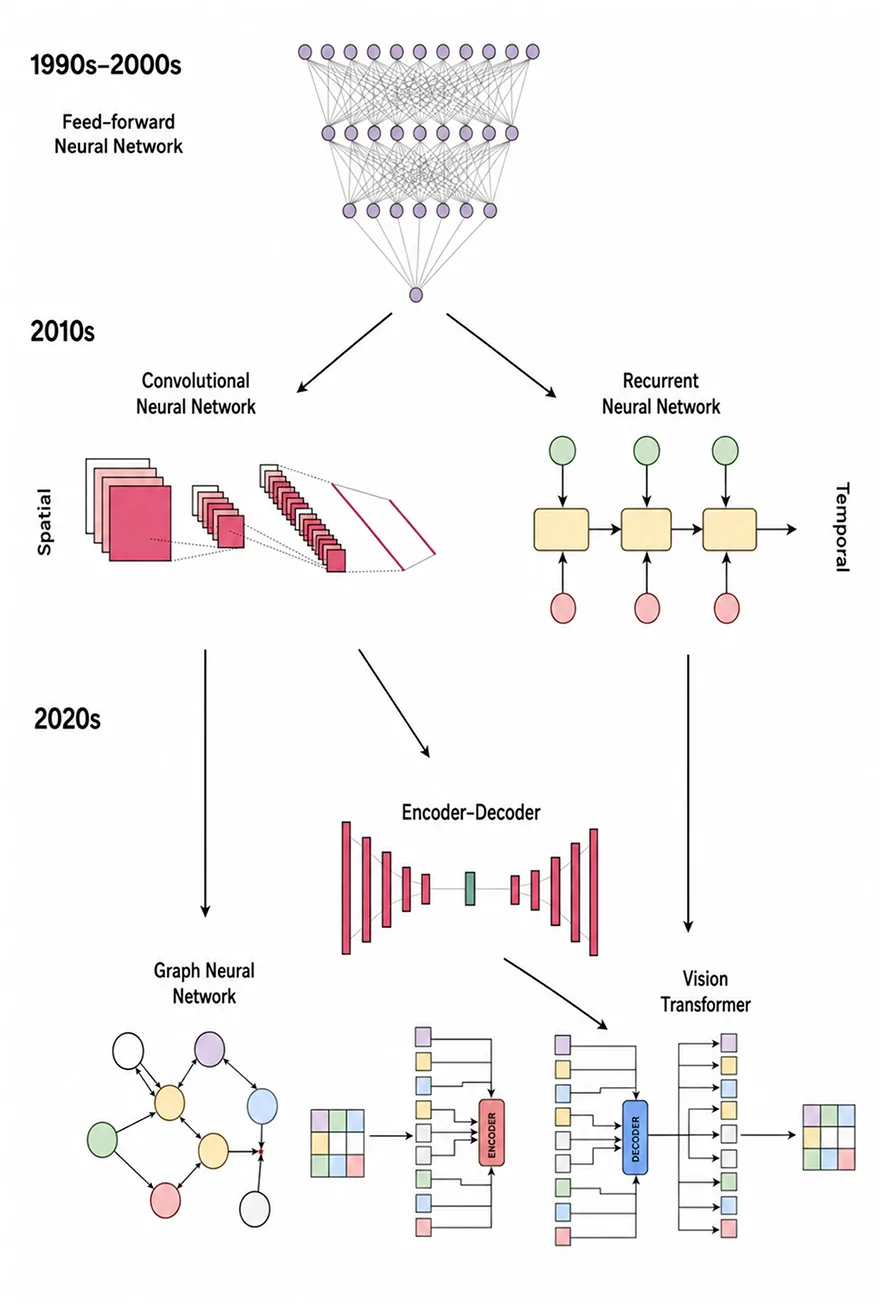
Evolution of rainfall prediction methodologies, illustrating the transition from empirical and statistical approaches to numerical weather prediction, machine learning, deep learning, and emerging foundation AI models for weather forecasting. Adapted and redrawn from Olivetti and Messori (2024).

Rainfall forecasting methodologies have evolved from empirical and statistical approaches to physically based NWP and, most recently, to AI-driven techniques. Early statistical models relied on historical rainfall data and assumed stationary climatic relationships, limiting their ability to capture the nonlinear and dynamic processes underlying ERE (Rouault and Richard, 2005; Wang et al., 2008; Holton, 2013; Doswell, 2001). Advances in atmospheric science and computational meteorology enabled a shift toward physically based modeling and, more recently, to data-driven learning paradigms. Figure 2 summarizes this methodological evolution and highlights the emergence of DL architectures for weather and rainfall prediction.

The introduction of NWP marked a significant advancement by enabling physically consistent simulations of atmospheric processes through the governing equations of fluid dynamics and thermodynamics. While NWP remains fundamental to operational forecasting, it still faces challenges with model parameterization, initialization, computational demands, and the accurate representation of localized convective processes that drive extreme rainfall (Bauer et al., 2015; Satyanarayana and Kar, 2016; Brotzge et al., 2023; Otero and Horton, 2023).

Recent advances in Earth observation systems, remote sensing, cloud computing, and high-performance computing have accelerated the adoption of AI-based forecasting methods. Unlike empirical or purely physics-based models, AI approaches learn complex nonlinear spatiotemporal relationships directly from diverse meteorological observations, including satellite imagery, radar data, and reanalysis datasets. As a result, these methods increasingly complement conventional forecasting systems and address several limitations inherent in traditional approaches (LeCun et al., 2015; Kim et al., 2017; Hernández et al., 2016; Waqas et al., 2025).

Recent advances in rainfall forecasting reflect a shift from viewing physics-based and AI approaches as competing paradigms to supporting integrated forecasting frameworks. These frameworks combine the physical consistency of NWP with the pattern recognition capabilities of DL. Hybrid systems improve the representation of complex atmospheric processes, enhance predictive skill across multiple forecasting horizons, and deliver more operationally relevant forecasts for ERE (Bauer et al., 2015; Bentivoglio et al., 2025; Zheng K. et al., 2024). The following subsection examines the scientific and technological factors driving the widespread adoption of AI for ERP.

### 3.5 Motivation for artificial intelligence in extreme rainfall prediction

The growing frequency and societal impacts of extreme rainfall have intensified demand for forecasting systems that are accurate, timely, and operationally reliable. Although NWP and traditional statistical approaches remain foundational to operational forecasting, these methods often fail to capture nonlinear atmospheric processes, multiscale interactions, and rapidly evolving convective events. Simultaneously, advances in Earth observation, weather radar, satellite platforms, and *in situ* sensing have generated unprecedented volumes of meteorological data, creating new opportunities for data-driven forecasting (Bauer et al., 2015; Ivancic and Shaw, 2016; Nash, 2017; Johnson et al., 2025).

Artificial Intelligence (AI), particularly machine learning (ML) and DL, has emerged as a complementary forecasting paradigm that learns complex spatio-temporal relationships directly from heterogeneous observations. By modeling nonlinear rainfall variability and spatio-temporal dependencies without relying exclusively on predefined physical parameterizations or statistical assumptions, AI addresses several persistent challenges in ERP (LeCun et al., 2015; Hernández et al., 2016; Gope et al., 2016; Waqas et al., 2025).

Advancements in high-performance computing, cloud infrastructure, and graphics processing units (GPUs), along with the availability of open environmental datasets, have further accelerated the operational adoption of AI. Concurrently, increased emphasis on disaster risk reduction and climate resilience has spurred the development of forecasting systems that integrate data-driven learning with physical knowledge to improve robustness, interpretability, uncertainty quantification, and operational reliability (Kim et al., 2017; Bentivoglio et al., 2025; Zheng K. et al., 2024; Trivedi et al., 2024b).

Together, these scientific and technological advances have established AI as a central component of contemporary rainfall forecasting, enabling the development of advanced spatio-temporal learning and operational prediction methodologies. The following section reviews these AI approaches and their contributions to ERP (Li C. et al., 2024; Fang et al., 2025; Li J. et al., 2024).

## 4 State-of-the-art artificial intelligence for extreme rainfall prediction

Accurate prediction of EREs is critical for effective early warning and disaster risk reduction (Gope et al., 2016; Byaruhanga et al., 2025). As thermodynamic warming increases atmospheric moisture, rainfall's heightened sensitivity to temperature underscores the need for adaptive predictive systems (Ivancic and Shaw, 2016; Satyanarayana and Kar, 2016).

In regions such as southern Africa, increasing hydro-climatic uncertainty has intensified the need for resilience planning (Nash, 2017; Johnson et al., 2025). Improving predictive capability directly enhances operational performance (Bauer et al., 2015), especially when leveraging data-driven methodologies. The DeepRain study (Kim et al., 2017) exemplifies this approach by seeking to reduce flood exposure and deliver actionable lead times (Bentivoglio et al., 2025).

Deep learning uses multi-layer neural networks to extract hierarchical representations from large, high-dimensional datasets (LeCun et al., 2015; Waqas et al., 2025). Unlike traditional approaches, DL approximates complex nonlinear functions across spatial and temporal domains, enabling the identification of latent structures directly from observational data (Hernández et al., 2016; Trivedi et al., 2024b).

Advances in recurrent model development enabled explicit temporal learning, with the introduction of Convolutional Long Short-Term Memory (ConvLSTM) networks (Shi et al., 2015) marking a foundational advance by incorporating convolutional operations into memory cells. Subsequent studies (Kim et al., 2017) advanced the field by integrating multichannel radar data and improving generative realism. Modern architectures, such as GFRNet (Fang et al., 2025) and Diffusion Transformers (Li J. et al., 2024), now accurately resolve high-intensity convective cores that were previously smoothed by standard regression models.

The evolution of DL for rainfall prediction reflects a shift from shallow representation learning to highly specialized spatiotemporal architectures. This progression was driven by the need to capture the nonlinear, multi-scale dynamics of precipitation systems, which traditional linear models could not adequately represent. Early DL adoption relied on Artificial Neural Networks (ANNs) and Multilayer Perceptron (MLP) architectures. Research (Gope et al., 2016; Hernández et al., 2016) shows that these foundational models improved nonlinear mappings between meteorological predictors and rainfall outcomes, outperforming classical statistical baselines. Nevertheless, these architectures treated atmospheric observations as independent feature vectors, overlooking essential spatial and temporal hierarchies.

A major methodological advance occurred with the adoption of Convolutional Neural Networks (CNNs), which maintained the spatial organization of radar and satellite imagery. The move toward hierarchical feature learning, as established by LeCun et al. (2015), enhanced the extraction of meso-scale structural features and storm morphology. However, although CNNs effectively captured spatial patterns, they lacked the capacity for temporal persistence. This shortcoming was initially addressed by RNNs and LSTM networks, which enabled the retention of storm history across time steps and substantially improved the modeling of rainfall persistence (Frame et al., 2021).

Convolutional Long Short-Term Memory (ConvLSTM) networks marked a significant breakthrough in rainfall prediction. In a seminal study, Shi et al. (2015) incorporated convolutional operators directly into recurrent memory cells. This advancement enabled the simultaneous learning of spatial storm structures and temporal transitions within a unified framework, laying the groundwork for modern nowcasting. Further refinements, such as integrating multichannel radar inputs in stacked layers, improved the tracking of intense rain cells (Kim et al., 2017).

By 2020, research priorities had shifted toward hybrid encoder-decoder architectures and the incorporation of attention mechanisms. Recent developments address multi-scale interactions, encompassing both local convective triggers and broader synoptic moisture transport (Zheng K. et al., 2024; Trivedi et al., 2024b). The evolution from nonlinear tabular learning to fully integrated spatiotemporal sequence modeling now characterizes the state of the art, enabling researchers to address the complexities of extreme meteorological events (Waqas et al., 2025; Li C. et al., 2024).

### 4.1 Evolution of AI methodologies for extreme rainfall prediction

**Figure 3:**
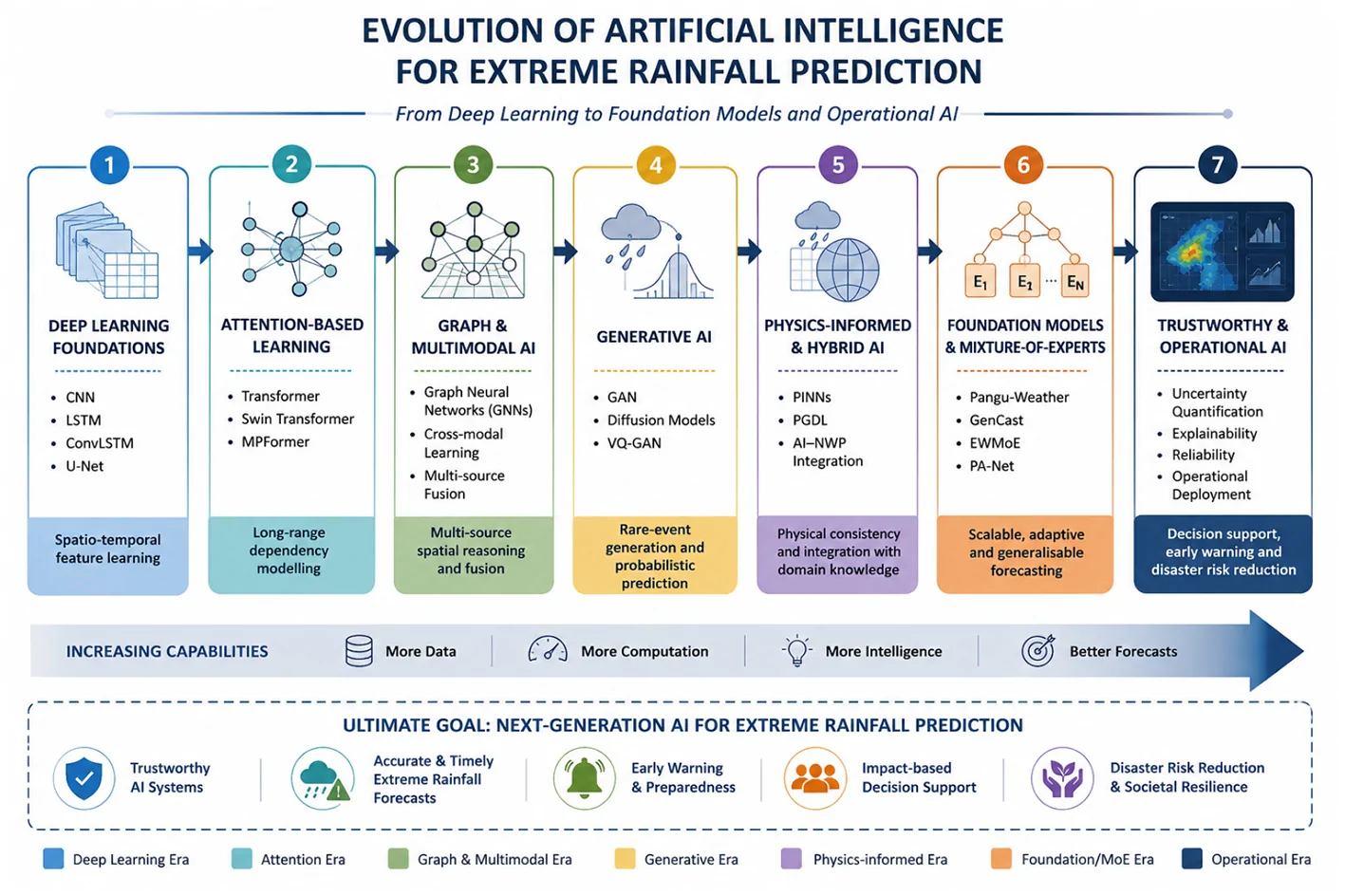
Evolution of Artificial Intelligence methodologies for extreme rainfall prediction. Conceptualized from literature synthesis and drawn by the authors.

Artificial Intelligence (AI) methodologies for ERP have evolved from early DL models focused on predictive accuracy to advanced forecasting systems that integrate heterogeneous environmental data, physical knowledge, uncertainty estimation, and operational decision support (LeCun et al., 2015; Waqas et al., 2025; Bentivoglio et al., 2025). Early research primarily used convolutional and recurrent neural networks to extract spatiotemporal rainfall patterns from historical observations (Kim et al., 2017; Hernández et al., 2016; Ren et al., 2021). As forecasting requirements expanded to include higher spatial resolution, longer lead times, and improved generalization, subsequent studies adopted attention mechanisms, Transformer-based architectures, graph neural networks, multimodal learning, and hybrid AI frameworks. These methods enable the integration of data from radar, satellite imagery, NWP, and *in situ* observations (Trivedi et al., 2024b; Zheng K. et al., 2024; Bauer et al., 2015). Figure 3 illustrates the progression from foundational DL architectures to current operational AI paradigms.

These advancements mark successive stages in the evolution of AI for rainfall forecasting, rather than mutually exclusive methodologies. The focus has gradually shifted from improving predictive accuracy to developing forecasting systems that are physically consistent, uncertainty-aware, scalable, and suitable for operational deployment. Recent paradigms, including generative AI, foundation models, MoE, and AI–NWP integration, further advance forecasting by improving scalability, supporting probabilistic prediction, and enabling the modeling of rare extreme events (Li C. et al., 2024; Fang et al., 2025; Li J. et al., 2024; Chen, 2025; Gan et al., 2025; Jiang et al., 2025; Xiao et al., 2026). Although Foundation Models and Mixture-of-Experts (MoE) architectures both aim to improve scalability and operational forecasting capabilities, they represent distinct AI paradigms. Foundation models emphasize large-scale pre-training and general-purpose representation learning, whereas MoE architectures employ adaptive expert routing and dynamic specialization for heterogeneous forecasting scenarios. Table 6 summarizes the principal stages of this evolution, outlining representative methodologies, primary capabilities, and key research challenges.

**Table 6:** Evolution of Artificial Intelligence methodologies for extreme rainfall prediction.

AI evolution stage	Representative methods	Primary capability	Remaining challenges
Foundational deep learning	CNN, LSTM, U-Net, ConvLSTM	Spatio-temporal feature extraction from historical meteorological observations	Generalization across climatic regions and rare extreme events
Attention-based learning	Transformer, Swin Transformer, MPFormer	Long-range temporal dependency modeling and global context learning	High computational complexity and data requirements
Graph-based & multimodal learning	Graph Neural Networks (GNNs), Cross-modal Learning	Spatial reasoning across multiple weather stations and heterogeneous environmental data	Data heterogeneity, graph construction, and scalability
Generative AI	GAN, Conditional GAN, Diffusion Models, VQ-GAN	Rare-event generation, probabilistic forecasting, and uncertainty-aware prediction	Calibration, hallucination, and forecast reliability
Physics-Informed & Hybrid AI	PINNs, PGDL, AI–NWP Integration	Integration of atmospheric knowledge with numerical weather prediction to improve physical consistency	Model complexity, optimization, and validation
Foundation models and expert-based AI	Pangu-Weather, GenCast (Foundation Models); EWMoE, PA-Net (Mixture-of-Experts)	Scalable large-scale weather forecasting through foundation-model pre-training and adaptive expert specialization.	Reliability, transferability, uncertainty quantification, and operational deployment

### 4.2 Foundational temporal deep learning models

Recurrent Neural Networks (RNNs) were the first DL models used for sequence-to-sequence learning in meteorology, enabling the analysis of precipitation changes over time from historical data. Their use in ERP has declined because of the vanishing gradient problem, which limits the modeling of long-term atmospheric trends needed for early detection of extreme events (Verma et al., 2023). This challenge has intensified in a warming climate, where increased atmospheric moisture, as described by the Clausius-Clapeyron relationship, produces complex rainfall patterns that basic RNNs cannot represent effectively (Ivancic and Shaw, 2016). Despite these limitations, basic recurrent models remain important benchmarks. Studies show that RNNs using detailed atmospheric data can produce reliable short-term forecasts (De Sousa Araújo et al., 2022). Additionally, recent research highlights a shift in rainfall forecasting toward integrating AI with NWP systems and diverse meteorological datasets (Trivedi et al., 2024a), reflecting ongoing advances in temporal DL for operational forecasting.

To overcome the limitations of standard RNNs, researchers have adopted LSTM models, which more effectively capture long-term dependencies in hydro-meteorological data. Unlike basic RNNs, LSTMs can learn persistent atmospheric processes that influence ERE. Notably, Frame et al. (2021) demonstrated that LSTM models often outperform traditional hydrological models, particularly when training data for extreme events are scarce. This advantage is especially evident in coastal and mountainous regions, where factors such as soil moisture, moisture transport, and prolonged rainfall play critical roles in the occurrence of floods and heavy precipitation (Patro and Bartakke, 2024; Patakchi Yousefi et al., 2024).

Recent studies suggest that combining LSTM architectures with deep feature extraction significantly improves models' capacity to represent complex, nonlinear rainfall processes that standard recurrent models often fail to capture (Devi et al., 2025; Tang et al., 2024). Further research on hybrid DL approaches indicates that future progress in rainfall forecasting will likely depend on integrating recurrent temporal learning with other DL architectures, rather than relying exclusively on LSTM models (Trivedi et al., 2026).

LSTM architectures have consistently outperformed other methods in rainfall forecasting across diverse hydro-meteorological applications. Since their introduction by Hochreiter and Schmidhuber (1997), studies have shown that LSTMs can model long-term atmospheric processes and nonlinear rainfall patterns that standard recurrent networks cannot capture. These strengths are especially important for capturing gradual meteorological changes, including moisture transport, geopotential height anomalies, and soil moisture persistence, all of which play significant roles in extreme precipitation (Frame et al., 2021; Patro and Bartakke, 2024; Afandi et al., 2025). The widespread use of LSTM models in rainfall forecasting is primarily due to their ability to learn complex temporal relationships, rather than solely to their internal gating mechanisms.

Extensive evidence supports the practical effectiveness of LSTM-based forecasting systems. For example, De Sousa Araújo et al. (2022) found that an LSTM model using twelve atmospheric reanalysis pressure levels produced accurate short-range forecasts, with a Mean Absolute Error of approximately 6.9 mm and a Root Mean Square Error of about 0.94 mm for six-hour predictions. Similarly, Kagabo et al. (2024) showed that LSTM models consistently outperformed CNN and GRU models on a multi-decade Rwandan rainfall dataset, even with noisy data. Another study (Kim and Kim, 2025) demonstrated that advanced LSTM variants more effectively captured evolving rainfall patterns. Recent hybrid recurrent frameworks have further improved forecasting accuracy by combining temporal sequence learning with deep feature extraction (Trivedi et al., 2026), highlighting the evolution of LSTM architectures toward more integrated forecasting systems.

Despite their proven effectiveness, LSTM architectures have significant limitations. They primarily capture temporal patterns in sequences of observations and typically operate on point-based or low-dimensional inputs, limiting their ability to model complex spatial rainfall patterns directly. This constraint is especially evident when forecasting convective systems, which depend on interactions among adjacent atmospheric regions rather than isolated points. Achieving optimal performance often requires extensive feature engineering and careful selection of meteorological predictors, raising concerns about the generalizability of these models across different climates (Patro and Bartakke, 2024). Yuan (2025) also found that purely recurrent models are insufficient to represent spatial rainfall variability unless combined with convolutional feature extraction. Recent studies suggest that improving model generalization requires architectures capable of learning both spatial and temporal rainfall patterns (Trivedi et al., 2024c). These challenges have led to the development of convolutional and hybrid DL models that incorporate spatial feature learning, which will be discussed in the following subsection.

### 4.3 Convolutional deep learning approaches for spatial rainfall representation

Convolutional Neural Networks (CNNs) are widely used DL architectures for spatial rainfall prediction because they automatically learn hierarchical precipitation features from gridded meteorological data. Unlike recurrent models, which primarily capture temporal dependencies, CNNs extract localized rainfall structures, such as convective cells, orographic gradients, and meso-scale precipitation patterns, from radar, satellite, and NWP products. This approach has substantially improved short-term rainfall forecasting while maintaining lower computational complexity than purely recurrent architectures (Ebtehaj and Bonakdari, 2024; Zhao et al., 2024). Recent advances have extended CNN-based rainfall forecasting beyond conventional convolution and pooling architectures by incorporating attention mechanisms, skip connections, and improved feature extraction strategies. Attention-augmented convolutional networks enhance representations of convective rainfall cores, while skip connections inspired by highway networks enable deeper architectures without compromising gradient propagation during training (Huang, 2022; Ren et al., 2021). However, research indicates that increasing network depth alone may over-smooth high-frequency precipitation structures and underestimate extreme rainfall intensity, underscoring that effective feature learning is more important than architectural complexity (Camps-Valls et al., 2025).

**Figure 4:**
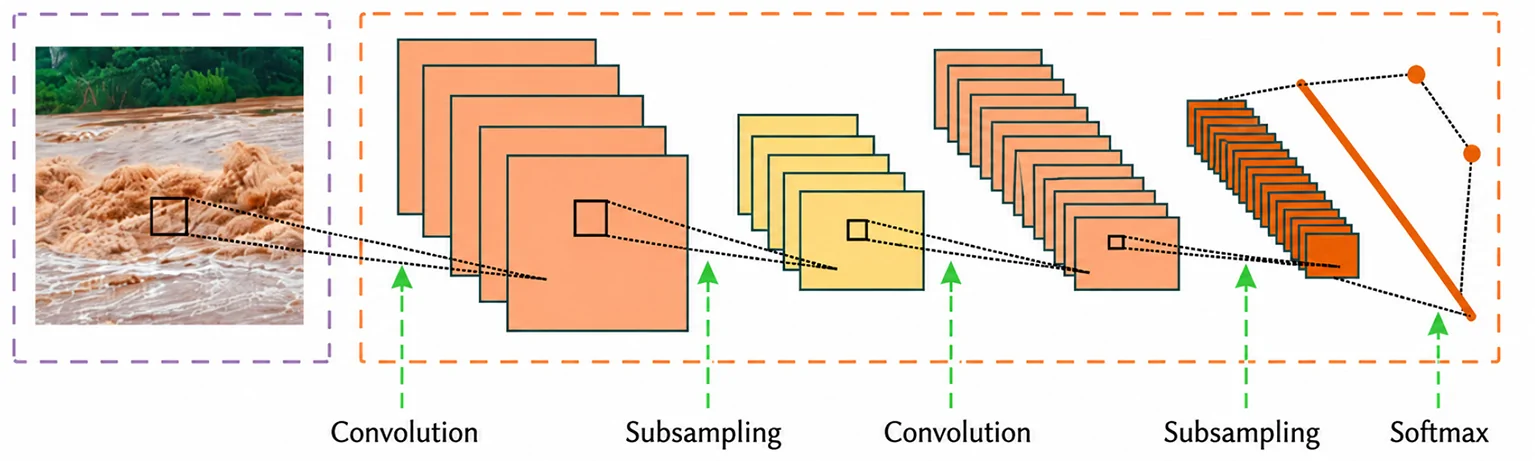
Structural diagram of a Convolutional Neural Network (CNN). Adapted from (Tan et al. 2024).

Further progress has been achieved through multimodal data fusion, which integrates radar, satellite imagery, NWP outputs, and topographic information within unified convolutional frameworks. Recent studies consistently report improved rainfall estimation, greater forecasting accuracy, and faster convergence when using multimodal CNN architectures. These results indicate that advancements increasingly depend on the effective integration of heterogeneous meteorological observations rather than on increasing network size (Cheng et al., 2025; Bui et al., 2025). Despite these advances, conventional CNNs remain limited in modeling long-range temporal dependencies and evolving atmospheric dynamics. These limitations have motivated the development of encoder-decoder architectures, particularly U-Net models, which integrate hierarchical spatial feature extraction with dense reconstruction of rainfall fields. The following subsection examines this progression. Figure 4 presents a representative CNN architecture.

### 4.4 Encoder–decoder architectures for dense rainfall field reconstruction

**Figure 5:**
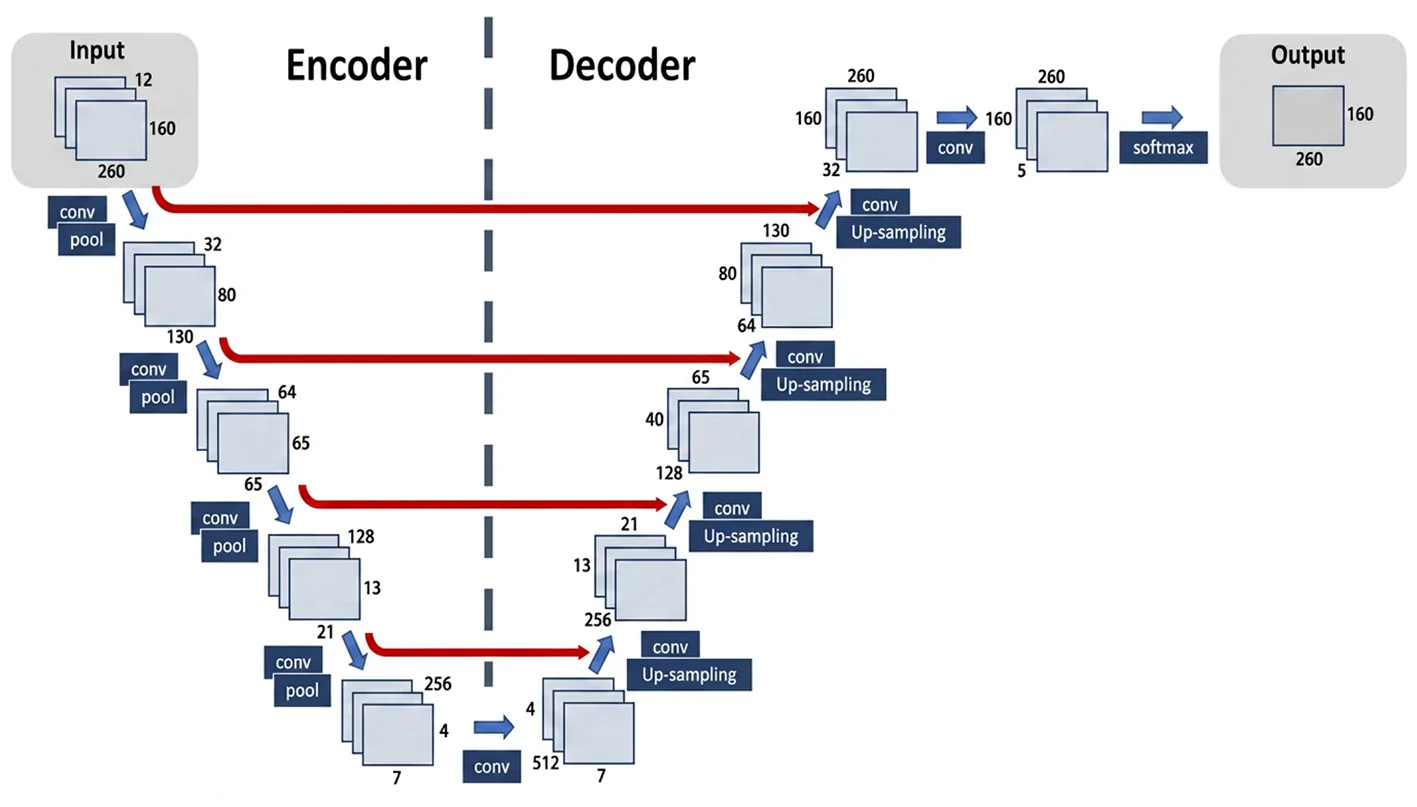
Symmetric U-Net architecture for precipitation nowcasting. The encoder **(left)** performs feature extraction and dimensionality reduction, while the decoder **(right)** utilizes kilometer-scale up-sampling and skip connections to reconstruct the rainfall field. Redrawn after being adapted from: Chen et al. (2023).

Encoder–decoder architectures, particularly U-Net, are essential to contemporary precipitation nowcasting because they reconstruct dense rainfall fields with high accuracy while preserving fine-scale spatial variability. Unlike conventional convolutional networks that compress spatial information, U-Net combines hierarchical feature extraction with multi-scale reconstruction via skip connections. This approach is highly effective for representing localized convective rainfall, radar echoes, and high-gradient precipitation patterns. Consequently, U-Net is widely used for quantitative precipitation estimation, rainfall nowcasting, and radar-based rainfall reconstruction. Figure 5 illustrates the general encoder–decoder framework used in these models.

Empirical studies consistently demonstrate the effectiveness of encoder–decoder architectures across a range of climatic conditions. U-Net-based systems outperform conventional NWP and other DL models while maintaining computational efficiency and robustness for ERP and kilometer-scale precipitation reconstruction (Fan et al., 2024; Otero and Horton, 2023; Sharma et al., 2023; Xu et al., 2026). Recent operational research suggests that further advancements are achieved primarily through multimodal data integration and improved feature learning, rather than increased architectural complexity. Despite these advancements, encoder–decoder architectures have limitations in representing long-range atmospheric interactions, which reduce forecasting skill as prediction horizons extend (Lyu et al., 2023). Their performance depends on data quality, observational representativeness, and the ability to generalize under increasingly non-stationary climatic conditions (Olivetti and Messori, 2024; Senjaliya et al., 2026). Furthermore, Frame et al. (2021) emphasize that highly accurate DL models require explicit physical constraints to ensure robustness under evolving atmospheric regimes. These challenges have motivated the development of transformer-based architectures, physics-informed learning, and hybrid spatio-temporal models that jointly capture local rainfall structures and broader atmospheric dynamics.

### 4.5 Transformer-based model architectures

**Figure 6:**
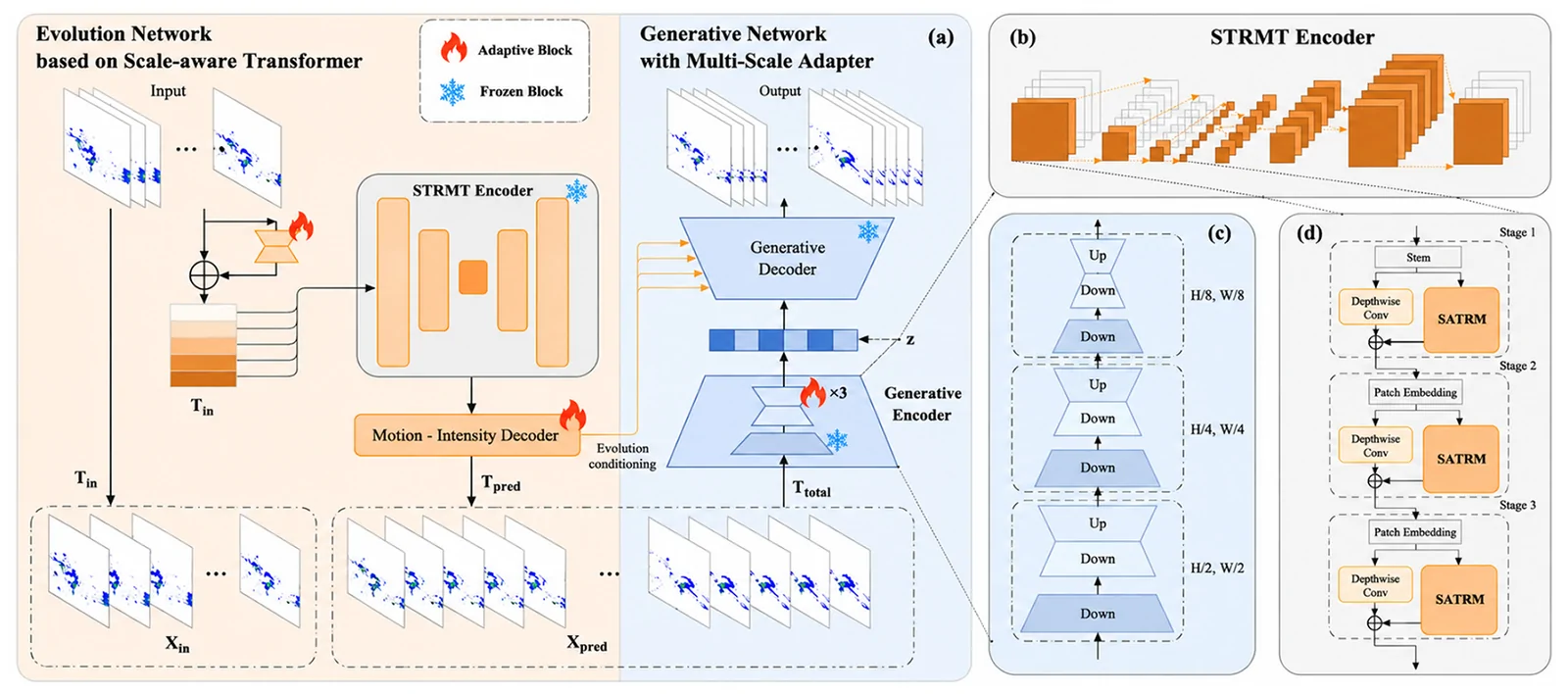
The MPFormer framework for precipitation nowcasting. **(a)** Overall architecture showing the dual-path Evolution and Generative Networks; **(b)** STRMT Encoder workflow; **(c)** Multi-Scale Adapter structure; **(d)** Detailed STRMT internal components. Adapted from: Zheng et al. (2025).

Transformer-based architectures mark a significant advance in DL for ERP, capturing long-range spatial and temporal dependencies that conventional recurrent or convolutional networks struggle to model. The self-attention mechanism enables modeling interactions among geographically distant atmospheric processes, making Transformers well-suited for precipitation nowcasting, seasonal rainfall forecasting, and multi-source meteorological data fusion. Early studies demonstrated the effectiveness of Temporal Fusion Transformers for extended-lead precipitation prediction. Recent research has incorporated convolutional feature extraction and generative learning to improve modeling of complex rainfall dynamics (Civitarese et al., 2021). Figure 6 illustrates a representative hybrid Transformer framework.

Recent studies show that progress in Transformer-based forecasting is driven primarily by integrating attention mechanisms with multi-scale feature extraction, generative learning, and task-specific optimization. For example, MPFormer combines attention-based temporal learning with multi-scale spatial feature extraction for precipitation nowcasting, while NowcastingGPT employs an Extreme Value Loss (EVL) objective to improve modeling of rare ERE (Zheng et al., 2025; Meo et al., 2024). These developments highlight a shift from stand-alone attention models to hybrid Transformer architectures for complex hydro-meteorological forecasting.

Operational evaluations further underscore the maturity of Transformer-based forecasting systems. Space-time Transformer architectures have achieved state-of-the-art performance in operational weather forecasting, and comparative studies consistently report improved modeling of convective storm evolution compared with earlier deep generative models and diffusion-based approaches (Harris and Chen, 2025; Franch et al., 2025b).

Nevertheless, their performance is constrained by high computational demands and the need for representative multi-regional training datasets, limiting operational deployment (Husain et al., 2025). Current evidence suggests that Transformer models are most effective when integrated with complementary DL paradigms, rather than used as stand-alone forecasting systems. Table 7 summarizes representative Transformer-based rainfall nowcasting studies.

**Table 7:** Recent Transformer-based nowcasting studies, data sources, and key findings.

Study	Model type	Data source	Key metrics & findings
GPTCast	GPT-style Transformer + VQGAN	Radar reflectivity	Probabilistic metrics (e.g., CRPS). Strong performance compared with ensemble extrapolation. Strong skill for high-intensity events.
SwinNowcast	Swin Transformer	KNMI radar	Evaluated using CSI, HSS, FAR. Strong performance at 5–10 mm thresholds.
Transformer Radar-from-Satellite	Encoder–decoder Transformer	Geostationary satellite	Reconstructs radar fields up to 2 hours ahead. Robust under rapidly evolving conditions.
Spatiotemporal Fusion Transformer	Squared-attention Transformer	Radar composites	Improves multi-step forecasting accuracy. Stronger performance for convective rainfall.
Diffusion Transformer	Diffusion + Transformer	Radar–satellite fusion	Captures long-range dependencies. Enhances spatial realism in forecasts.

### 4.6 Generative deep learning for extreme rainfall prediction

Generative DL enhances rainfall forecasting by explicitly modeling the probability distribution of precipitation fields, thereby surpassing deterministic prediction approaches. Rather than prioritizing average prediction accuracy, generative models reconstruct realistic rainfall distributions, effectively capturing localized convective structures and long-tailed precipitation extremes that conventional loss functions tend to smooth out (Harris et al., 2022). Consequently, these architectures are increasingly important for probabilistic forecasting, uncertainty-aware nowcasting, and improved representation of rare, high-impact rainfall events.

Recent studies consistently demonstrate the advantages of generative learning for extreme rainfall forecasting. Architectures such as GFRNet, vector-quantized GANs with Transformer decoders, and multimodal ConvLSTM-GAN frameworks achieve improved reconstruction of long-tailed rainfall distributions, convective rainfall cores, and sharp intensity gradients relative to deterministic forecasting models (Fang et al., 2025; Yin et al., 2024; Lu et al., 2024). Collectively, these results suggest that the principal benefit of generative learning lies in producing more realistic rainfall fields with explicit representation of forecasting uncertainty. Recent research has introduced hybrid generative architectures that integrate convolutional, recurrent, diffusion, and probabilistic learning components. Notable examples include CLGAN, which combines U-Net architectures, recurrent learning, and three-dimensional discriminators; CasCast, which uses latent diffusion for probabilistic rainfall forecasting; and physics-informed generative frameworks that improve forecast realism and interpretability (Ji et al., 2022; Gong et al., 2024; Taghizadeh et al., 2025). Operational studies further demonstrate the growing use of generative models as post-processing frameworks for NWP, thereby improving the reconstruction of localized rainfall extremes from coarse-resolution forecasts (Xu et al., 2026). Despite these advancements, generative models still face challenges, including unstable adversarial optimization, mode collapse, and reliance on representative training data and physically meaningful constraints (Ren et al., 2021). These limitations have spurred greater interest in diffusion-based generative models, which offer more stable probabilistic learning while preserving spatial realism and uncertainty representation. The following subsection examines these emerging diffusion architectures.

### 4.7 Diffusion models and probabilistic rainfall forecasting

Diffusion models mark a significant advance in generative DL for ERP. They enable stable probabilistic learning, supporting ensemble rainfall forecasting and explicit representation of forecast uncertainty. By reconstructing rainfall fields from stochastic latent representations, diffusion models improve characterization of extreme precipitation and consistently outperform conventional convolutional architectures in probabilistic rainfall forecasting. Recent studies report notable gains in forecasting accuracy and uncertainty representation, including up to 15% performance gains over U-Net-based systems and improved reconstruction of rare, high-intensity precipitation events (Li C. et al., 2024; Meo et al., 2024).

Despite these advances, probabilistic diversity alone is insufficient for reliable operational forecasting. As noted by Khot et al. (2024), uncertainty estimates must be carefully calibrated before probabilistic predictions can effectively support EWS and operational decision-making. Consequently, future research should focus not only on generating diverse rainfall realizations but also on producing physically meaningful and statistically reliable uncertainty estimates appropriate for risk-informed forecasting.

### 4.8 Hybrid deep learning architectures

Recent advances in DL for ERP increasingly prioritize hybrid architectures because individual neural network paradigms cannot adequately capture the complexity of atmospheric processes. Modern forecasting systems incorporate temporal, spatial, probabilistic, and physically informed learning strategies within unified frameworks to leverage the strengths of each model and mitigate their limitations. Hybrid architectures do not constitute a distinct model family; instead, they integrate recurrent, convolutional, attention-based, and generative approaches, providing a versatile foundation for robust spatio-temporal rainfall forecasting across varied meteorological contexts.

#### 4.8.1 CNN–transformer hybrid architectures

CNN–Transformer hybrid architectures have been developed to overcome the limitations of convolutional and attention-based models by jointly learning localized rainfall structures and long-range atmospheric interactions. This approach is particularly effective for multi-source meteorological forecasting, which requires integrating heterogeneous observations across multiple spatial and temporal scales. Empirical studies show that combining convolutional feature extraction with attention mechanisms significantly improves precipitation forecasting performance compared with single-stream architectures. For example, the CNN-BiLSTM-AM model incorporates convolutional encoders, bidirectional recurrent learning, and attention mechanisms to leverage precipitable water vapor (PWV) and Convective Available Potential Energy (CAPE) for rainfall prediction. Additional cross-modal frameworks further underscore the benefits of multimodal feature fusion (Zheng K. et al., 2024; Tang and Gui, 2023; Zheng Q. et al., 2024).

In addition to CNN–Transformer integration, encoder–decoder hybrid frameworks have advanced rainfall forecasting by incorporating NWP bias correction, weighted-loss optimization, multi-predictor learning, and attention-guided spatial feature extraction. Recent research consistently demonstrates enhanced forecasting performance with U-Net-based hybrid architectures, especially in data-scarce regions and areas with complex terrain, where accurately representing localized rainfall extremes remains difficult (Sharma et al., 2023; Lyu et al., 2023; Fan et al., 2024; Kwon et al., 2023; Trivedi et al., 2024b). Overall, these results indicate that hybrid forecasting systems achieve optimal benefits by integrating complementary learning paradigms with diverse meteorological observations.

#### 4.8.2 Integration of artificial intelligence and numerical weather prediction

The integration of DL with NWP has emerged as a prominent strategy for operational rainfall forecasting. This approach combines physically consistent atmospheric simulations with AI-driven bias correction, downscaling, and uncertainty refinement. Rather than replacing numerical forecasting systems, DL enhances predictive skill by post-processing model outputs. Recent studies show that DL-based post-processing consistently improves precipitation forecasts, reduces systematic prediction errors, and yields more realistic heavy rainfall forecasts than conventional statistical correction methods (Rojas-Campos et al., 2023; Lee et al., 2025).

Recent advances have expanded the integration of AI and NWP through diffusion-based post-processing and neural operator architectures. These techniques improve the spatial realism of precipitation forecasts and learn systematic corrections directly from numerical model outputs (Liu et al., 2025; Wiegels et al., 2026). When combined with multimodal hybrid frameworks, these approaches show that integrating data-driven learning with physically based forecasting yields more accurate representations of fine-scale precipitation while preserving the physical consistency of NWP simulations (Qi et al., 2026; Wiegels et al., 2026; Humphries et al., 2026).

Despite these advances, DL cannot fully resolve deficiencies arising from inaccurate initial conditions or imperfect physical parameterizations in numerical weather models. Consequently, the effectiveness of AI-assisted forecasting fundamentally depends on the quality of meteorological inputs and the physical realism of the underlying NWP system (Wang et al., 2023).

### 4.9 Physics-informed deep learning

While DL has substantially improved rainfall forecasting, purely data-driven models may produce predictions that contradict established atmospheric and hydrological principles. These inconsistencies are especially problematic during ERE, as physically implausible forecasts can undermine operational decision-making and EWS. Physics-Informed Deep Learning (PIDL) addresses this challenge by embedding physical knowledge directly into the learning process. Rather than treating physical modeling and AI as competing methodologies, PIDL integrates observational data with established scientific principles to improve forecast realism, generalization, and interpretability across diverse hydro-climatic conditions.

Physical knowledge can be incorporated into DL models through mechanisms such as conservation-law constraints, physically meaningful loss functions, integration of NWP outputs, and the inclusion of atmospheric variables that describe moisture transport, energy balance, and precipitation dynamics. Empirical studies indicate that maintaining physically consistent rainfall–runoff relationships enhances model robustness under changing environmental conditions, while embedding physical constraints in generative rainfall forecasting improves both forecast realism and interpretability (Frame et al., 2021; Taghizadeh et al., 2025).

Beyond improving predictive accuracy, physics-informed learning addresses a fundamental limitation of purely data-driven forecasting: limited generalization to conditions not represented in the training data. Comprehensive assessments by Camps-Valls et al. (2025) and Olivetti and Messori (2024) indicate that progress in Earth system AI depends on integrating observational learning with established scientific understanding, rather than merely increasing model complexity. This integration is especially relevant for ERP, where evolving climatic conditions, non-stationary weather regimes, and sparse observations of rare events pose significant challenges for conventional ML models.

Current challenges include formulating appropriate physical constraints, balancing observational and physical objectives during optimization, and maintaining computational efficiency while improving generalization across diverse climatic environments. Addressing these challenges has led to the development of adaptive learning frameworks, such as MoE architectures, that dynamically allocate specialized prediction strategies based on prevailing meteorological conditions.

### 4.10 Mixture-of-experts architectures

Mixture-of-Experts (MoE) architectures address the limitations of single-model DL systems, which often lack generalizability across climatic regimes, rainfall intensities, and forecasting horizons. Instead of using a single global model, MoE frameworks dynamically allocate specialized neural networks to distinct forecasting scenarios. This approach enables more accurate modeling of heterogeneous rainfall processes and enhances the flexibility and scalability of forecasting models.

Recent studies have demonstrated that adaptive expert routing is increasingly effective for precipitation forecasting. Notable frameworks, including the Precipitation-Adaptive Network (PA-Net) and Knowledge-Guided Adaptive Mixture-of-Experts, dynamically route rainfall regimes to specialized expert networks and incorporate meteorological knowledge into routing. These methods improve modeling of long-tail rainfall events and enhance forecast interpretability (Xiao et al., 2026; Jiang et al., 2025).

In addition to regional precipitation forecasting, MoE architectures have been applied to global weather prediction. The EWMoE framework shows that adaptive allocation of specialized forecasting modules improves predictive performance across diverse meteorological regimes, underscoring the scalability of expert-based learning for operational weather forecasting (Gan et al., 2025). Collectively, these advances position MoE architectures as a progression from traditional hybrid models toward more adaptive and scalable AI forecasting systems.

Ongoing research challenges include managing computational complexity, achieving effective expert specialization, ensuring routing stability, and assembling sufficiently diverse training datasets to prevent expert collapse and redundancy. Addressing these challenges is expected to facilitate the integration of MoE with GNN, physics-informed learning, and foundation models, thereby establishing a flexible foundation for next-generation intelligent weather forecasting systems.

Although Mixture-of-Experts architectures share certain scalability characteristics with emerging foundation models, they are primarily distinguished by adaptive expert routing and dynamic specialization rather than by large-scale pre-training on general-purpose datasets. Table 8 summarizes the current state of the art by consolidating representative studies, data sources, pre-processing strategies, and model architectures reported in the literature.

**Table 8:** Representative advances in Artificial Intelligence for extreme rainfall prediction.

Study	AI paradigm	Primary innovation	Data sources	Key contribution
Frame et al. (2021)	DL	Mass-conserving LSTM	CAMELS, meteorological forcing, streamflow	Improved hydrological consistency and extrapolation capability.
Choi and Kim (2023)	Spatio-temporal Learning	ConvLSTM trajectory modeling	Radar, ASOS observations	Enhanced learning of evolving precipitation structures.
Fan et al. (2024)	Multimodal Learning	Multi-predictor U-Net	ERA5, ECMWF, satellite observations	Fusion of heterogeneous environmental predictors for high-resolution rainfall forecasting.
Zheng K. et al. (2024)	Cross-modal AI	Joint multimodal representation learning	Radar, satellite, rain gauges	Improved information fusion across complementary sensing modalities.
Xu et al. (2026)	Generative AI	Conditional GAN	ECMWF outputs, observations	Improved post-processing of precipitation forecasts and extreme rainfall representation.
Harris and Chen (2025)	Generative AI	Transformer with Extreme Value Loss	Weather4Cast radar benchmark	Tail-aware learning for improved prediction of rare extreme rainfall events.
Fang et al. (2025)	Hybrid AI	Generative fusion residual network	Radar mosaics, NWP outputs	Improved spatial reconstruction through AI–NWP integration.
Chen (2025)	Physics-informed AI	Physics-informed Transformer	Global climate and reanalysis datasets	Integration of physical constraints into DL frameworks.
Jiang et al. (2025)	Mixture-of-Experts	Knowledge-guided Adaptive MoE	Multimodal meteorological observations	Adaptive expert routing for heterogeneous precipitation prediction.
Gan et al. (2025)	Mixture-of-Experts	EWMoE	ERA5 global weather data	Scalable Mixture-of-Experts framework for operational weather forecasting.
Xiao et al. (2026)	Mixture-of-Experts	PA-Net	ERA5 precipitation datasets	Adaptive expert allocation for long-tail rainfall nowcasting.

## 5 Prediction horizons and operational intelligence

### 5.1 Operational perspectives across forecasting horizons

Forecast accuracy and prediction horizon jointly determine the operational value of ERP. The prediction horizon sets the decision-making context, the availability of meteorological data, the dominant atmospheric processes, and the choice of evaluation metrics. Different lead times serve distinct objectives, including real-time emergency response to rapidly evolving convective storms and long-term planning for water resource management and climate adaptation. Therefore, the suitability of a DL model should be evaluated within its defined operational context.

The lack of a universal definition of extreme rainfall complicates comparisons across forecasting studies. Recent research uses various criteria, including fixed rainfall thresholds, percentile-based measures, meteorological warning levels, and flood-trigger thresholds. Each criterion affects class imbalance, model development, evaluation metrics, and the interpretation of forecast skill, as discussed in Section 3.

As the prediction lead time increases, the primary sources of predictability shift from radar and satellite observations to NWP, large-scale atmospheric circulation, teleconnections, and slowly varying climate drivers. Consequently, each forecasting horizon requires specialized DL approaches rather than a universal forecasting architecture. The following subsections review recent advances in DL for nowcasting, short-range forecasting, and subseasonal-to-seasonal (S2S) prediction. These sections examine the dominant AI paradigms, operational strengths, current limitations, and emerging research directions.

### 5.2 Artificial intelligence approaches for rapid convective storm nowcasting (0 to 6 h)

Nowcasting is a well-established operational application of AI in meteorology, addressing the challenge of resolving rapidly evolving convective storms that NWP alone cannot adequately capture. Deep learning models use high-frequency radar, satellite, and lightning observations to generate timely forecasts that support flash-flood warnings, emergency response, aviation safety, and urban flood management. Reviews by Olivetti and Messori (2024) and Camps-Valls et al. (2025) identify this forecasting horizon as particularly well-suited to data-driven learning because it can directly capture evolving storm structures from observations.

ConvLSTM architectures are the leading approach for precipitation nowcasting because they model both spatial rainfall patterns and temporal evolution. Recent studies show that integrating ConvLSTM models with imbalance-aware learning strategies, such as weighted-loss optimization and stratified sampling, significantly improves detection of high-intensity rainfall and outperforms conventional nowcasting systems at 10- to 60-min lead times (Choi et al., 2025a; Wang Y. V. et al., 2024).

Generative and probabilistic AI models are expanding the capabilities of operational nowcasting. Transformer-based models trained with event-based learning improve predictions of rare, high-intensity precipitation events. In parallel, adversarial ConvLSTM frameworks enhance the reconstruction of convective rainfall cores from multisource satellite observations (Bi et al., 2023; Lu et al., 2024). Diffusion-based methods further advance probabilistic nowcasting by generating rainfall ensembles that explicitly quantify forecast uncertainty (He G. et al., 2025; Husain et al., 2025).

Despite these advancements, operational deployment still faces challenges, including the rapid decline in forecast skill with increasing lead time, sensitivity to training data, and limited observations of rare extreme events. Consequently, current research focuses on multimodal data fusion, adaptive learning, uncertainty quantification, and physically informed AI to enhance the robustness of operational EWS.

### 5.3 Short-range forecasting (1–4 days): integrating hybrid artificial intelligence in operational weather prediction

Short-range forecasting bridges observation-driven nowcasting and extended climate prediction. For lead times of one to four days, precipitation is primarily governed by synoptic-scale circulation, moisture transport, frontal dynamics, and orographic forcing. Integrating DL with NWP enables physically consistent simulations, data-driven bias correction, spatial refinement, and improved representation of high-impact rainfall. Recent reviews highlight the integration of AI and NWP as a central direction for operational weather forecasting (Dotse et al., 2024; Ejike et al., 2025; Olivetti and Messori, 2024; Camps-Valls et al., 2025). Hybrid DL models consistently improve NWP forecasts through post-processing. U-Net-based approaches correct systematic model biases while preserving fine-scale precipitation structures, yielding more accurate predictions of extreme rainfall and enhanced performance in complex terrain and heterogeneous environments (Sharma et al., 2023; Trivedi et al., 2024b; Rahimi et al., 2024; Lyu et al., 2023).

Recent advances extend hybrid forecasting beyond conventional bias correction by integrating multimodal observations, physically derived atmospheric predictors, and probabilistic learning into operational decision-support systems. Studies show that combining physical model outputs with data-driven learning improves forecast robustness for high-impact rainfall events and provides a basis for physics-informed, uncertainty-aware operational forecasting (Gope et al., 2016). Persistent challenges include dependence on NWP simulation quality, limited transferability across diverse climatic regions, and insufficient robustness in uncertainty quantification. Addressing these issues drives the development of physically consistent hybrid architectures, adaptive learning strategies, and more comprehensive integration of AI within operational forecasting workflows.

### 5.4 Subseasonal-to-seasonal forecasting: artificial intelligence for climate-scale predictability

Subseasonal-to-seasonal (S2S) forecasting is challenged by the rapid decline in atmospheric predictability beyond synoptic timescales. Unlike shorter forecasting horizons, S2S prediction depends primarily on slowly varying climate drivers, large-scale atmospheric circulation, and teleconnection patterns such as the El Niño–Southern Oscillation (ENSO), the Madden–Julian Oscillation (MJO), and monsoon variability. Consequently, S2S forecasting requires AI models capable of learning long-range spatial and temporal dependencies in highly non-stationary climatic environments. Reviews by Olivetti and Messori (2024) and Camps-Valls et al. (2025) identify this as a primary direction for next-generation Earth system AI. Transformer-based architectures, neural operators, and hybrid models have shown significant promise for S2S forecasting. Recent studies demonstrate improved weekly to monthly prediction accuracy by integrating long-range temporal learning with large-scale atmospheric predictors, thereby outperforming conventional seasonal forecasting methods (Civitarese et al., 2021; Lyu et al., 2023; Fan et al., 2024). These findings underscore the importance of integrating complementary atmospheric information over extended forecasting horizons.

Recent advances have expanded S2S forecasting capabilities through adaptive Fourier Neural Operators (AFNOs) and foundation models, which improve seasonal bias correction, spatial coherence, and the use of global Earth observation datasets (Wiegels et al., 2026). Collectively, these innovations are moving S2S forecasting toward scalable Earth system AI capable of representing complex atmospheric dynamics across multiple spatial scales. Persistent challenges include non-stationary climate variability, shifting teleconnection patterns, limited observations of rare extreme events, and insufficient transferability across climatic regions. Addressing these issues is driving research into physics-informed AI, uncertainty-aware forecasting, adaptive learning, foundation models, and graph-based representations to support climate-resilient EWS and long-term disaster preparedness.

## 6 Artificial intelligence across geographic and climatic contexts

### 6.1 Tropical monsoon convective systems

Tropical monsoon environments pose significant challenges for ERP because of the interplay among convective instability, seasonal monsoon circulation, complex terrain, and heterogeneous rainfall patterns across spatial and temporal scales. Therefore, effective AI models must accurately represent both localized convective processes and broader atmospheric forcing while remaining robust under variable observational conditions. Empirical studies indicate that multi-scale convolutional, attention-based, and RNN architectures improve rainfall prediction in tropical monsoon regions by capturing complementary spatial and temporal features of convection. For example, U-Net and attention-based models have demonstrated improved predictive performance across complex terrain in Assam and other monsoon environments. Broader evaluations identify multi-scale feature learning as particularly effective in regions with pronounced spatial variability in rainfall (Sharma et al., 2023; Trivedi et al., 2024b; Rahimi et al., 2024; Zhou et al., 2025; Camps-Valls et al., 2025).

In tropical Africa, limited observational coverage continues to constrain model development. Despite this limitation, recurrent DL models have produced promising rainfall predictions using long-term station data in Rwanda. However, reported classification accuracies should be interpreted cautiously, as severe class imbalance can inflate performance metrics for rare ERE (Kagabo et al., 2024). These findings support recommendations to evaluate tropical rainfall forecasting with tail-sensitive verification metrics rather than relying solely on overall accuracy (Wani et al., 2024; Ejike et al., 2025).

In summary, effective AI-based forecasting in tropical monsoon climates depends on model architecture, the quality of observational data, and the ability to generalize across diverse monsoon systems. Future advancements will require integrating multimodal observations, uncertainty-aware learning, and physics-informed AI to enable robust operational forecasting of high-impact tropical rainfall.

### 6.2 Mid-latitude convective systems

Mid-latitude convective systems pose substantial forecasting challenges because precipitation arises from interactions among frontal systems, mesoscale convection, topographic effects, and rapidly evolving atmospheric instabilities. Addressing these complexities requires AI models that capture both localized convective processes and broader atmospheric organization while remaining robust across a range of weather regimes. As a result, mid-latitude environments constitute a critical benchmark for evaluating model generalization.

Research consistently shows that encoder–decoder, hybrid convolutional, and regime-aware learning approaches improve rainfall prediction by preserving fine-scale precipitation structures and incorporating large-scale atmospheric information. Notably, U-Net-based models have improved rainfall-extreme prediction and achieved superior spatial downscaling, outperforming conventional seasonal forecasting systems in heterogeneous environments (Lyu et al., 2023; Fan et al., 2024; Zhao et al., 2024; Ren et al., 2021). These findings underscore the importance of multi-scale feature integration rather than reliance on specific network architectures.

Regime-aware learning strategies further improve forecasting performance in dynamically evolving mid-latitude environments. ConvLSTM-based models consistently outperform traditional nowcasting systems during warm-season convective events. Furthermore, clustering-guided training improves predictions of underrepresented extreme rainfall regimes and promotes generalization across diverse weather conditions (Wang Y. V. et al., 2024; Kwon et al., 2023; Olivetti and Messori, 2024; Choi and Kim, 2023).

Generative and hybrid AI models further advance these capabilities by improving predictions over complex terrain and producing more realistic representations of storm initiation and evolution. Conditional Latent GANs have demonstrated improved precipitation forecasting in mountainous regions, while FuXi Nowcast provides more realistic convective evolution than operational numerical forecasting systems for short-range prediction (Ji et al., 2022; Chen et al., 2025). These developments indicate a transition toward hybrid forecasting systems that combine physically based atmospheric knowledge with probabilistic AI.

In summary, effective forecasting in mid-latitude environments demands AI systems that adapt to diverse synoptic regimes rather than optimizing exclusively for a single weather type. Significant challenges persist, including model transferability, uncertainty quantification, and reliable prediction of rare extreme precipitation events. Overcoming these challenges is expected to accelerate the integration of physics-informed learning, multimodal Earth observations, and adaptive expert architectures for operational forecasting in highly variable atmospheric conditions.

### 6.3 Urban flash flood applications

Extreme rainfall prediction in urban environments is challenging because of high population density, extensive impervious surfaces, aging drainage infrastructure, and limited response times, all of which increase flood risk. Unlike regional forecasting, urban applications require AI models that provide reliable, high-resolution forecasts with sufficient lead time to inform emergency response, traffic management, and public warning systems. Therefore, the operational value of urban forecasting depends on both predictive accuracy and timely decision support.

Recent research shows that DL models effectively support neighborhood-scale urban flood forecasting. Stacked auto-encoders have accurately predicted extreme rainfall in Mumbai and Kolkata with lead times of 6 to 48 h. Many apparent false alarms corresponded to rainfall in adjacent catchments, thereby extending the operational warning area (Gope et al., 2016). These findings suggest that modest over-prediction is acceptable in urban contexts, where the consequences of missing extreme events typically outweigh those of false alarms (Kim and Kim, 2025; Dotse et al., 2024).

Operational performance has improved through the adoption of recurrent neural network architectures and the integration of multimodal data sources. Long Short-Term Memory (LSTM) models have demonstrated competitive short-range forecasting performance in highly urbanized basins in southeastern Brazil. Additionally, integrating radar observations with citizen-science rain-gauge reports significantly improves forecasting accuracy in areas with limited conventional monitoring networks (De Sousa Araújo et al., 2022; Rosenhoover et al., 2025). These studies underscore the importance of incorporating heterogeneous observational data to achieve reliable urban flood prediction. Recent advances are steering urban forecasting toward uncertainty-aware, impact-focused decision support. Weighted-loss ConvLSTM models improve detection of rare, high-impact rainfall events by addressing class imbalance. Accurate forecasts with 10- to 60-min lead times improve preparedness for drainage overflow and roadway flooding during rapidly evolving storms (Choi et al., 2025a; Wang S. et al., 2024). Broader evaluations indicate that operational urban forecasting increasingly relies on high-resolution probabilistic guidance that communicates forecast uncertainty alongside rainfall intensity (Camps-Valls et al., 2025; He S. et al., 2025).

In summary, effective urban flash-flood forecasting requires accurate rainfall prediction and the translation of forecasts into actionable risk information. Continued progress will depend on integrating multimodal observations, adaptive learning, graph-based representations of urban drainage networks, and physics-informed AI to support resilient EWS in smart cities.

## 7 Key innovations and comparative analysis

**Figure 7:**
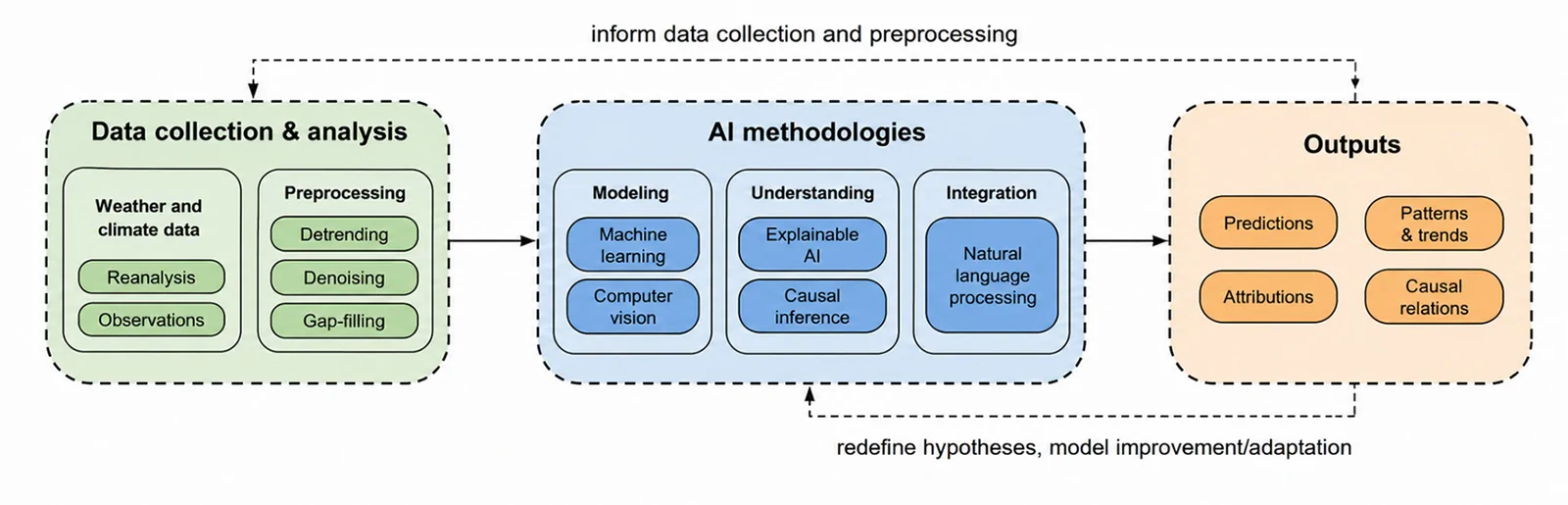
A common pipeline for Artificial Intelligence-driven extreme event analysis. Redrawn after adaptation from Camps-Valls et al. (2025).

Previous sections examined AI applications for ERP from methodological, operational, and geographic perspectives. This section synthesizes the principal innovations that have advanced the field, with particular emphasis on event-aware learning, multimodal data fusion, and robust evaluation strategies. Collectively, these developments facilitate the transition from conventional DL models to operationally reliable and scientifically trustworthy forecasting systems. Figure 7 presents a conceptual synthesis of the methodological workflow underlying contemporary AI-driven ERP. The framework illustrates the progression of heterogeneous atmospheric observations through preprocessing, multimodal feature integration, DL model development, performance evaluation, and operational forecasting applications. Rather than depicting a specific architecture, it summarizes the common methodological pipeline identified in the reviewed literature.

The framework highlights the increasing integration of heterogeneous observations, advanced learning paradigms, and rigorous evaluation strategies that characterize modern AI-based extreme rainfall forecasting.

### 7.1 Learning strategies for rare extreme events

Extreme rainfall prediction faces fundamental limitations due to severe class imbalance, as high-impact precipitation events occur far less often than light or moderate rainfall. Consequently, conventional optimization objectives often prioritize common rainfall events, reducing model sensitivity to rare extremes. Recent research has increasingly focused on event-aware learning strategies that explicitly emphasize the upper tail of rainfall distributions during model optimization. Reviews by Camps-Valls et al. (2025); Ren et al. (2021); Olivetti and Messori (2024) identify this transition as a principal methodological advance in DL for ERP. Weighted-loss optimization remains the most widely adopted strategy for improving detection of rare rainfall events across ConvLSTM, U-Net, Transformer, and hybrid forecasting systems (Choi et al., 2025b; Lyu et al., 2023; Fan et al., 2024). Recent approaches have extended this paradigm by introducing distribution-aware and physics-informed learning objectives that preserve convective intensity and balance predictive accuracy with physical consistency (Chen et al., 2025; Dotse et al., 2024). Collectively, these developments reflect a broader shift from static weighting schemes to adaptive, uncertainty-aware, and physically constrained optimization strategies for operational extreme rainfall forecasting.

### 7.2 Multimodal data fusion in operational forecasting

Extreme rainfall results from atmospheric processes that occur across multiple spatial and temporal scales, which are not fully captured by any single observing system. As a result, contemporary AI methods increasingly employ multimodal data fusion to integrate complementary information from radar, satellite imagery, NWP, reanalysis products, and surface observations. Modeling interactions among heterogeneous atmospheric variables enables these approaches to improve predictions of rapidly evolving convective storms and other high-impact precipitation events. Reviews by Zhou et al. (2025); Yu Z. et al. (2025); Camps-Valls et al. (2025) identify multimodal learning as a defining characteristic of next-generation operational rainfall forecasting. Empirical studies consistently demonstrate that integrating complementary Earth observation datasets yields superior forecasting performance compared to single-source approaches. The combination of satellite observations, radar data, NWP outputs, terrain information, and atmospheric reanalysis improves the representation of convective rainfall across diverse climatic environments (Lu et al., 2024; Harris and Chen, 2025; Sharma et al., 2023; Trivedi et al., 2024b; De Sousa Araújo et al., 2022). These findings indicate that predictive improvements are primarily due to the exploitation of complementary atmospheric information, rather than merely increasing data volume. Recent forecasting systems advance multimodal learning by incorporating physics-aware feature interactions, which surpass traditional data fusion techniques. Unified frameworks such as FuXi and MAG-Net demonstrate that jointly modeling relationships among heterogeneous atmospheric observations improves convective representation, physical consistency, and operational forecasting performance (Chen et al., 2025, 2026). Similar results are observed for GPTCast and interpretable multimodal architectures, indicating that physically meaningful feature representations enhance both forecast generalization and interpretability across diverse weather regimes (Franch et al., 2025a; Taghizadeh et al., 2025). In summary, multimodal data fusion has evolved from a data integration technique to a foundational design principle for operational AI-based rainfall forecasting. Future progress is expected to rely on adaptive fusion mechanisms, foundation models trained on heterogeneous Earth observation datasets, and uncertainty-aware, physics-informed representations that improve forecast robustness, physical consistency, and decision support for EWS.

### 7.3 Evaluation, benchmarking and operational verification

Reliable evaluation is essential in AI for ERP because no single performance metric fully captures forecasting skill for rare, high-impact events. Current benchmarking practices combine continuous, categorical, probabilistic, and operational metrics to assess rainfall magnitude, event detection, forecast uncertainty, and the value of decisions. Reviews by Olivetti and Messori (2024); Camps-Valls et al. (2025); Yu H. et al. (2025) highlight that comprehensive evaluation is necessary for meaningful comparison of DL models. Continuous regression metrics measure the agreement between predicted and observed rainfall amounts. However, these metrics are relatively insensitive to rare extreme events because non-extreme rainfall cases dominate the dataset. As a result, categorical verification metrics are commonly used to evaluate the detection of hazardous precipitation, providing a more balanced assessment of forecasting skill across both common and extreme rainfall regimes (Choi et al., 2025b; Fan et al., 2024; Chen et al., 2025).

The adoption of probabilistic forecasting has broadened evaluation beyond deterministic accuracy measures. Metrics such as the Continuous Ranked Probability Score (CRPS), Brier Score, and calibration diagnostics assess forecast confidence and reliability alongside predictive accuracy. These metrics are particularly valuable for operational forecasting of rare ERE (Khot et al., 2024; Meo et al., 2024). This trend underscores the growing importance of uncertainty-aware AI systems for risk-informed decision support.

Benchmarking has evolved from simple accuracy assessment to multidimensional evaluation that incorporates predictive performance, uncertainty, operational usefulness, and forecast reliability.

Additional verification techniques further strengthen the evaluation of probabilistic rainfall forecasts. Probability Integral Transform (PIT) histograms provide a complementary assessment of calibration for continuous predictive distributions, while ensemble spread-skill relationships assess whether forecast uncertainty aligns with observed prediction errors. The conventional Continuous Ranked Probability Score (CRPS) evaluates the overall predictive distribution, whereas threshold-weighted probabilistic verification metrics place greater emphasis on rare, high-impact rainfall events by assigning additional weight to specified regions of the predictive distribution. From an operational perspective, cost-loss and relative economic value analyses quantify the practical benefits of probabilistic forecasts for EWS by balancing the consequences of false alarms and missed detections.

Future benchmarking frameworks are expected to prioritize calibration, explainability, uncertainty quantification, and impact-oriented verification. These advances will facilitate more transparent comparison of AI systems across diverse climatic regions and operational contexts. Table 9 summarizes the main evaluation categories used in ERP, along with their primary purposes and limitations.

**Table 9:** Evaluation frameworks commonly used in Artificial Intelligence for extreme rainfall prediction.

Evaluation category	Representative metrics	Primary purpose	Limitation if used alone
Continuous accuracy	RMSE, MAE, NSE	Quantify errors in predicted rainfall magnitude and overall regression performance.	Limited sensitivity to rare and high-intensity rainfall events.
Event detection	CSI, POD, FAR, ETS	Assess the ability to detect hazardous and extreme rainfall events.	Does not quantify forecast uncertainty or confidence.
Probabilistic verification	CRPS, Brier Score, Reliability Diagram, Calibration Curve	Evaluate forecast confidence, uncertainty, and probabilistic reliability.	Requires probabilistic forecasting models or ensemble predictions.
Operational verification	Lead Time, Warning Success Rate, Decision-support Metrics	Assess the practical usefulness of forecasts for early warning and operational decision-making.	Highly application- and stakeholder-dependent.

## 8 Application domains and operational deployment

Previous sections examined AI for ERP from methodological, forecasting, geographic, and comparative perspectives. This section evaluates how these advances have been implemented in operational contexts, including EWS, flood forecasting, disaster risk management, and climate resilience. Together, these applications demonstrate the progression of AI from experimental research to a practical decision-support tool for addressing complex hydro-meteorological hazards.

### 8.1 Early warning systems

Early warning systems (EWS) are a critical operational application of AI, as even modest improvements in forecast lead time can significantly reduce flood impacts and enhance emergency preparedness. The effectiveness of these systems depends on predictive accuracy, reliable communication of uncertainty, and the timely translation of meteorological information into actionable decisions. Empirical studies show that DL substantially enhances early warning capabilities when adapted to local meteorological conditions. Hybrid forecasting systems that combine NWP with high-resolution DL improve detection of orographically driven extreme rainfall in complex terrain. These systems provide valuable operational guidance in regions dominated by local convection (Sharma et al., 2023; Trivedi et al., 2024b). Operational deployment has been implemented in densely populated urban environments, where rapid flood development limits response time. Stacked autoencoder models have accurately identified extreme rainfall over Mumbai and Kolkata, with lead times of 6 to 48 h. Many apparent false alarms were linked to neighboring catchments, effectively extending the operational warning region. Related studies indicate that integrating diverse environmental observations improves situational awareness in areas with limited conventional urban monitoring networks (Gope et al., 2016; Bui et al., 2025). In data-limited environments, recurrent DL models provide effective support for flash-flood early warning. LSTM-based forecasting has shown robust performance in Rwanda's flood-prone regions. Integrating convolutional and recurrent architectures enhances the representation of spatial rainfall patterns and temporal storm evolution under noisy observational conditions (Kagabo et al., 2024; Ebtehaj and Bonakdari, 2024). These findings underscore the importance of aligning AI models with local hazard characteristics and data availability.

AI-driven EWS are evolving from rainfall-forecasting tools into integrated decision-support platforms that integrate probabilistic predictions, uncertainty communication, and real-time environmental observations. Continued advancements are expected to enhance the operational reliability and societal value of AI-assisted disaster risk reduction amid increasingly variable climatic conditions.

### 8.2 Flood forecasting and disaster management

Flood forecasting is a critical operational application of AI because effective disaster management depends on translating rainfall forecasts into reliable hydrological guidance. Unlike rainfall nowcasting, flood forecasting requires comprehensive predictions of precipitation, runoff generation, and inundation risk. Recent research increasingly integrates DL with hydrological models, NWP, and decision-support frameworks to enhance flood preparedness amid rapidly changing hydro-meteorological conditions.

Hybrid AI–NWP frameworks provide a robust foundation for operational flood forecasting by combining physically consistent atmospheric guidance with data-driven bias correction. These approaches reduce the propagation of forecast uncertainty across the flood prediction chain and improve performance under highly non-stationary rainfall conditions (Dotse et al., 2024; Nguyen et al., 2025). Furthermore, integrating satellite-derived precipitation products with DL corrections improves the spatial representation of extreme rainfall, thereby increasing the reliability of downstream flood forecasts. Operational flood forecasting is improved by learning strategies that enhance the detection and continuity of high-impact events. Weighted-loss learning increases sensitivity to intense precipitation, which drives rapid runoff generation. Accurate short-lead forecasts strengthen flash-flood warning capabilities, while DL-based rainfall infilling maintains forecasting continuity when observations are incomplete (Choi et al., 2025b; Wani et al., 2024; Meuer et al., 2024). Collectively, these advancements enhance the robustness of operational warning systems during rapidly evolving flood events. Recent advances increasingly integrate DL into hydrological forecasting workflows to support basin-scale flood prediction. Hybrid precipitation–runoff models, graph-based watershed representations, and CNN–LSTM architectures demonstrate that combining meteorological prediction with hydrological response yields more reliable flood guidance across diverse catchments (Mandraha, 2024; Salcedo, 2024; Ebtehaj and Bonakdari, 2024). These developments indicate a growing convergence between meteorological forecasting and hydrological impact modeling. Flood forecasting is shifting from isolated rainfall prediction to integrated hydro-meteorological decision-support systems. Continued progress will require closer integration of AI, physically based hydrological models, uncertainty quantification, graph-based watershed representations, and impact-based forecasting frameworks that deliver transparent, actionable flood-risk information for emergency managers.

### 8.3 Climate change impact assessment

Climate change poses substantial challenges for ERP by altering the frequency, intensity, spatial distribution, and temporal patterns of precipitation extremes. Atmospheric warming affects moisture availability, storm dynamics, and large-scale circulation patterns, which may diminish the predictive skill of AI models trained exclusively on historical observations in non-stationary climatic contexts. Consequently, recent research prioritizes developing AI systems that maintain reliability under evolving climate regimes and support climate resilience, adaptation planning, and long-term hydro-meteorological risk assessment.

Recent studies indicate that DL methods can detect climate-driven changes in extreme rainfall and augment conventional climate modeling frameworks. Advanced temporal and spatial learning techniques improve the representation of evolving precipitation regimes, seasonal variability, and shifting monsoon dynamics under non-stationary climatic conditions (Civitarese et al., 2021; Kwon et al., 2023; Trivedi et al., 2024b). Collectively, these findings indicate that AI can capture complex climate-related precipitation patterns that are often difficult to represent with conventional statistical approaches alone. Beyond forecasting, AI supports climate impact assessment by correcting systematic biases in dynamical climate simulations and improving the representation of extreme precipitation. Deep learning bias-correction techniques and multimodal learning frameworks strengthen regional climate projections by integrating complementary observational and modeling data (Lyu et al., 2023; Fan et al., 2024; Zhou et al., 2025). These advancements provide a more robust foundation for regional climate projections and Earth system simulations, where accurately representing extreme rainfall remains a significant scientific challenge. Future progress will depend on AI systems that remain robust under non-stationary climatic conditions, preserve physical consistency, and provide reliable uncertainty estimates. Advances in transfer learning, physics-informed neural networks, foundation models, and uncertainty-aware learning are expected to improve AI's transferability across climatic regions and strengthen its role in climate adaptation, infrastructure planning, and long-term disaster resilience.

## 9 Discussion

### 9.1 Synthesis of findings

The reviewed evidence shows that AI for ERP has evolved from isolated DL architectures to integrated forecasting ecosystems. These ecosystems integrate complementary modeling paradigms, diverse environmental observations, and physically informed learning strategies. Recent advances stem from integrating recurrent, convolutional, attention-based, generative, and physics-guided approaches, each tailored to specific aspects of the forecasting problem. This progression underscores the growing complexity of predicting high-impact hydro-meteorological extremes and highlights a shift toward methodological integration rather than architectural replacement (Olivetti and Messori, 2024; Camps-Valls et al., 2025; Yu H. et al., 2025).

A second key finding is that forecasting performance is strongly shaped by the interplay among model design, prediction horizon, atmospheric processes, and the availability of observations. Short-term nowcasting primarily benefits from ConvLSTM, Transformer, and generative architectures, which effectively represent rapidly evolving convective systems. Conversely, short-range forecasting increasingly uses hybrid AI and NWP frameworks that combine physical consistency with data-driven correction. At S2S timescales, predictive skill relies more on attention mechanisms, large-scale atmospheric predictors, and multimodal learning that can exploit teleconnection-driven predictability (Civitarese et al., 2021; Lyu et al., 2023; Wiegels et al., 2026).

The review also shows that recent advances increasingly prioritize operational deployment over benchmark accuracy alone. In EWS, flood forecasting, and climate impact assessment, successful implementation requires integrating heterogeneous environmental observations, uncertainty-aware prediction, and impact-oriented decision support. As a result, AI has advanced beyond rainfall estimation to enable comprehensive hydro-meteorological intelligence systems that support emergency preparedness, flood risk management, and climate adaptation (Camps-Valls et al., 2025; He S. et al., 2025; Husain et al., 2025).

Several methodological principles consistently emerge throughout the reviewed literature. Event-aware optimization improves learning from highly imbalanced rainfall datasets, multimodal data fusion strengthens the representation of complex atmospheric processes, and hybrid modeling enhances both generalization and physical consistency. More recent developments, including foundation models, adaptive MoE frameworks, uncertainty-aware learning, and physics-informed AI, further reinforce the shift toward integrated, interpretable, and operationally reliable forecasting systems (Khot et al., 2024; Jiang et al., 2025; Xiao et al., 2026; Gan et al., 2025).

In summary, the literature suggests that the future of AI for ERP will hinge on adaptive, trustworthy, and physically consistent forecasting systems. These systems will integrate environmental knowledge with data-driven learning to support decision-making across diverse climatic regions and forecasting horizons. This progression reflects a broader shift from architecture-centered research to integrated Earth system intelligence, in which advances in ML are increasingly informed by physical understanding, uncertainty quantification, and societal needs (Olivetti and Messori, 2024; Camps-Valls et al., 2025; Franch et al., 2025b).

### 9.2 Current limitations and research challenges

Although DL has led to significant advances in ERP, operational deployment remains constrained by inadequate generalization across climatic regions, forecasting horizons, and observational networks. These limitations stem from atmospheric non-stationarity, sparse observational data, class imbalance, high computational requirements, and limited integration of physical knowledge into data-driven models. Therefore, improvements in predictive accuracy alone do not ensure operational reliability in forecasting systems (Olivetti and Messori, 2024; Camps-Valls et al., 2025; Yu H. et al., 2025). Model generalization remains a primary barrier to operational deployment. While many studies demonstrate strong performance within specific climatic regions, predictive skill frequently diminishes when models are applied to different hydro-meteorological environments. This decline reflects variations in observation density, rainfall climatology, topography, and sensor characteristics. These challenges underscore the necessity for domain adaptation, transfer learning, and the creation of geographically diverse benchmark datasets to ensure robust performance across heterogeneous environmental conditions (Franch et al., 2025b; Pasche et al., 2025; Wani et al., 2024). Establishing trustworthy AI for operational forecasting remains challenging. Although many models achieve high benchmark accuracy, few provide well-calibrated uncertainty estimates, interpretable outputs, or transparent evaluations that support operational decision-making. As AI becomes more integrated into early warning and disaster management systems, uncertainty quantification, explainability, calibration, and rigorous validation are as critical as predictive accuracy. This is especially true for high-impact rainfall events, where both missed detections and false alarms can have substantial societal consequences (Khot et al., 2024; Husain et al., 2025; Camps-Valls et al., 2025). Emerging approaches, including foundation models, adaptive MoE architectures, neural operators, and physics-informed learning, offer promising strategies to overcome current limitations. These methods may enhance scalability, transferability, and physical consistency. Furthermore, closer integration of AI with NWP could improve both predictive skill and scientific interpretability. However, systematic evaluation of these techniques across diverse climatic regions remains scarce, highlighting a critical area for future research (Jiang et al., 2025; Xiao et al., 2026; Wiegels et al., 2026). In summary, the literature suggests that advancing robust, transferable, uncertainty-aware, and physically consistent forecasting systems is more critical than further increasing neural network complexity. Addressing these challenges will require enhanced collaboration across atmospheric science, hydrology, AI, and disaster risk management to develop forecasting frameworks that are scientifically credible, operationally viable, and societally beneficial (Olivetti and Messori, 2024; Camps-Valls et al., 2025; He S. et al., 2025).

## 10 Research roadmap and future directions

### 10.1 Next-generation intelligent forecasting architectures

Future advancements in ERP are expected to rely less on identifying a single optimal DL architecture and more on developing adaptive forecasting systems that dynamically integrate complementary modeling paradigms in response to evolving atmospheric conditions. Synthesized evidence demonstrates that convolutional, recurrent, Transformer-based, generative, and hybrid approaches each offer distinct advantages across different rainfall regimes, forecasting horizons, and observational settings. Consequently, adaptive model selection and ensemble learning are emerging as critical strategies for enhancing forecasting robustness and reducing dependence on any single architecture (Olivetti and Messori, 2024; Camps-Valls et al., 2025).

Adaptive ensemble strategies effectively leverage the complementary strengths of multiple DL models. Recent research shows that dynamic model selection and ensemble optimization improve forecasting performance across diverse rainfall characteristics and prediction horizons by adapting to changing atmospheric conditions, rather than relying on a single forecasting approach (Choi et al., 2025b; An et al., 2024). These findings suggest that future operational systems will increasingly employ adaptive ensembles that integrate spatial, temporal, and contextual representations within unified forecasting frameworks. Dynamic expert routing represents a significant advancement in next-generation forecasting systems. Mixture-of-Experts architectures enable specialized subnetworks to address distinct meteorological conditions, thereby improving the representation of heterogeneous rainfall regimes while maintaining computational efficiency. Recent studies indicate that precipitation-adaptive expert routing, knowledge-guided expert selection, and expert-specialized global weather forecasting enhance the prediction of rare, high-intensity events and improve model interpretability and scalability (Xiao et al., 2026; Jiang et al., 2025; Gan et al., 2025). Foundation models trained on large-scale Earth observation datasets represent another promising avenue for future rainfall forecasting. These models enable transfer learning across climatic regions and support rapid adaptation to data-sparse environments, reducing reliance on region-specific training datasets and supporting more scalable, transferable forecasting systems (Camps-Valls et al., 2025; He S. et al., 2025). In summary, next-generation forecasting systems are expected to evolve into adaptive, physically informed, and transferable learning ecosystems that integrate dynamic model selection, expert specialization, foundation models, and Earth system knowledge within unified operational frameworks. These systems offer a promising approach to achieving more reliable hydro-meteorological forecasting across diverse climatic regions and environmental conditions (Olivetti and Messori, 2024; Franch et al., 2025b).

### 10.2 Physics-informed, hybrid, and climate-resilient artificial intelligence for rainfall prediction

Future advancements in ERP are expected to arise from integrating the predictive flexibility of DL with the foundational physical principles governing atmospheric processes. Although data-driven models exhibit strong predictive capabilities, they often lack physical consistency when applied beyond the environmental conditions represented in their training data or under non-stationary climatic regimes. Consequently, future research is expected to focus on forecasting systems that combine physical understanding with adaptive ML, thereby enhancing both predictive performance and scientific credibility (Camps-Valls et al., 2025; Olivetti and Messori, 2024; Yu H. et al., 2025).

Physics-informed learning offers a promising approach by embedding atmospheric constraints directly into model development. Rather than relying solely on statistical optimization, these methods enforce physically meaningful relationships during training. This approach reduces implausible predictions and improves robustness under changing climatic conditions (Frame et al., 2021; Taghizadeh et al., 2025).

Hybrid AI–NWP systems represent a significant direction for future operational forecasting. In this context, DL augments NWP by correcting systematic biases, enhancing spatial resolution, and improving the representation of extreme rainfall. The development of neural operator architectures further advances this integration by enabling the learning of physically meaningful mappings between atmospheric states across multiple spatial and temporal scales. Together, these advances suggest that future forecasting systems will increasingly integrate numerical simulation and AI within unified operational frameworks, rather than treating them as separate methodologies (Rojas-Campos et al., 2023; Lee et al., 2025; Wiegels et al., 2026).

The challenges posed by climate change underscore the need for adaptive, climate-resilient AI. As rainfall extremes evolve under non-stationary climatic conditions, forecasting systems must maintain transferability across regions, adapt continuously to new observations, and leverage diverse Earth observation datasets while preserving previously acquired knowledge. These capabilities are critical for supporting climate adaptation, resilient infrastructure planning, and long-term disaster risk management (Phillips et al., 2024; Yu Z. et al., 2025; Camps-Valls et al., 2025). In summary, the next generation of rainfall forecasting systems will likely be defined not by greater neural network complexity but by the ability to integrate physical realism, adaptive learning, and operational reliability within unified Earth-system forecasting frameworks. Achieving this vision will require strengthened collaboration among atmospheric science, hydrology, climate modeling, and AI to develop forecasting systems that are scientifically credible, operationally effective, and resilient to changing climatic conditions (Olivetti and Messori, 2024; Franch et al., 2025b; He S. et al., 2025).

### 10.3 Ensuring trustworthy artificial intelligence in operational forecasting

As AI transitions from experimental research to operational weather forecasting, future advancements should emphasize not only predictive accuracy but also the development of systems that are reliable for high-stakes decision-making. Extreme rainfall warnings directly influence emergency response, evacuation planning, infrastructure management, and disaster risk reduction, underscoring that forecast reliability is as critical as forecast skill. Consequently, operational AI must provide scientifically credible, transparent, and well-calibrated forecasts that can be confidently integrated into meteorological warning services (Camps-Valls et al., 2025; Olivetti and Messori, 2024; He S. et al., 2025). Explainability is essential for establishing trustworthy operational forecasting. Although modern DL models often outperform conventional methods, their internal decision-making processes remain opaque. Future forecasting systems should offer transparent explanations of the atmospheric variables, spatial patterns, and temporal processes that influence prediction outcomes. This level of transparency facilitates scientific validation, increases forecaster confidence, and supports informed decision-making during high-impact weather events (Taghizadeh et al., 2025; Husain et al., 2025; Camps-Valls et al., 2025). Quantifying and communicating predictive uncertainty is also critical. Future operational systems should distinguish between epistemic uncertainty, arising from model limitations, and aleatoric uncertainty, arising from inherent atmospheric variability. These systems must generate calibrated probabilistic forecasts, ensuring that predicted probabilities match observed event frequencies. Therefore, forecast evaluation should extend beyond deterministic accuracy to include probabilistic verification and calibration diagnostics that better reflect the operational value of forecasts for EWS (Khot et al., 2024; Meo et al., 2024; Franch et al., 2025b). Trustworthy forecasting depends on effective collaboration between AI and human expertise, rather than on replacing meteorological judgment. Future decision-support systems should integrate AI guidance with NWP, observational data, and expert interpretation. These systems must also provide calibrated uncertainty estimates to help forecasters balance false alarms and missed extreme events in line with operational risk requirements (Lee et al., 2025; Rojas-Campos et al., 2023; Camps-Valls et al., 2025). In summary, the long-term success of AI in extreme rainfall forecasting depends not only on predictive performance but also on explainability, physical consistency, uncertainty awareness, and operational reliability. Trustworthy AI should be established as a core design principle to ensure that advances in ML yield reliable forecasting systems that strengthen disaster preparedness, climate resilience, and public safety (Olivetti and Messori, 2024; Camps-Valls et al., 2025; He S. et al., 2025).

### 10.4 Operational deployment and decision support

Despite significant progress in DL methods for ERP, integration into routine meteorological operations remains limited. This limited adoption underscores the rigorous demands of operational forecasting, including computational efficiency, robustness, scalability, sustained reliability, and predictive accuracy. Therefore, future research must prioritize operational readiness by developing forecasting systems that meet both scientific standards and real-time operational requirements (Camps-Valls et al., 2025; Olivetti and Messori, 2024; He S. et al., 2025).

Operational deployment will likely focus on integrating AI into existing meteorological infrastructure rather than replacing conventional forecasting systems. Hybrid AI and NWP workflows, together with diverse Earth observation systems such as weather radar, satellite imagery, IoT sensor networks, and automated quality-control frameworks, provide complementary environmental data that enable continuous, resilient forecasting. These integrated forecasting ecosystems are expected to improve the robustness, spatial coverage, and responsiveness of operational warning services (Lee et al., 2025; Rojas-Campos et al., 2023; Wiegels et al., 2026). The principal aim of operational AI extends beyond rainfall prediction to include actionable decision support. Future forecasting systems should offer impact-oriented guidance through probabilistic hazard assessments, flood-risk indicators, and integrated multi-hazard forecasting that combines meteorological, hydrological, and environmental data within unified decision-support frameworks. These capabilities are expected to enhance emergency management, flood forecasting, transportation planning, water resource management, and disaster risk reduction (Dotse et al., 2024; Salcedo, 2024; Camps-Valls et al., 2025). Recent advances in cloud, edge, and high-performance computing, together with digital twin technologies, provide the technological foundation for scalable operational forecasting. These platforms support continuous model updates, rapid assimilation of diverse environmental observations, and near-real-time deployment across regional and national forecasting centers. As a result, they enable adaptive forecasting in response to evolving atmospheric and climatic conditions (Franch et al., 2025b; Wiegels et al., 2026; He S. et al., 2025). The success of operational deployment will depend on both institutional collaboration and algorithmic innovation. Enhanced partnerships among meteorological agencies, hydrologists, AI researchers, emergency managers, and policymakers are essential to developing forecasting systems that are reliable, interpretable, computationally efficient, and operationally sustainable. Bridging the divide between research innovation and routine forecasting practice will ultimately determine the long-term societal value of AI for ERP (Olivetti and Messori, 2024; Camps-Valls et al., 2025; Yu H. et al., 2025).

### 10.5 Key community challenges and research priorities

Although methodological advances in AI for ERP have been rapid, progress is increasingly constrained by fragmented evaluation practices that hinder objective comparisons of forecasting approaches. Differences in datasets, forecasting horizons, rainfall thresholds, verification metrics, and experimental protocols complicate consistent assessment of methodological improvements. Future research must prioritize reproducible experimentation, standardized evaluation frameworks, and collaborative research practices to enable robust comparisons across diverse forecasting methodologies (Olivetti and Messori, 2024; Camps-Valls et al., 2025; Yu H. et al., 2025). The development of openly accessible benchmark datasets that represent diverse climatic regions, precipitation regimes, and observational environments is a critical priority. Standardized benchmarks, together with harmonized verification protocols that incorporate deterministic, event-based, probabilistic, spatial, and uncertainty-aware evaluation, will enable more rigorous assessment of forecasting systems. These measures will also enhance reproducibility and facilitate meaningful comparison across future AI approaches (Franch et al., 2025b; Pasche et al., 2025; He S. et al., 2025). Open science should serve as the foundation for future research. Public access to datasets, source code, trained models, and reproducible workflows will accelerate methodological innovation and enhance transparency and scientific credibility. Sustained collaboration among atmospheric scientists, hydrologists, computer scientists, remote sensing specialists, emergency managers, and operational forecasting agencies is essential to ensure that methodological advances address real-world forecasting challenges rather than focusing exclusively on benchmark performance (Camps-Valls et al., 2025; Olivetti and Messori, 2024; Husain et al., 2025). Addressing the geographical imbalance in AI applications for ERP is critical. Current research is concentrated in regions with dense observational networks and advanced computational infrastructure, while many highly vulnerable areas remain underrepresented. Expanding international collaboration, strengthening local capacity, and promoting transferable forecasting frameworks and shared environmental data resources are essential to ensure that advances in AI benefit diverse climatic and socioeconomic contexts (Pasche et al., 2025; Franch et al., 2025b; Wani et al., 2024). In conclusion, sustained advances in AI for ERP depend on collaborative, reproducible, and globally inclusive research practices, as well as ongoing methodological progress. Emphasizing open science, rigorous benchmarking, interdisciplinary partnerships, and representative evaluation protocols will accelerate the development of robust, generalizable, and operationally deployable forecasting systems that support disaster risk reduction and enhance climate resilience (Olivetti and Messori, 2024; Camps-Valls et al., 2025; He S. et al., 2025).

## 11 Conclusions

This review synthesized recent advances in AI and DL for ERP, with particular emphasis on methodological developments reported from 2020 to 2026. Recent evidence indicates a progression from isolated DL models to integrated forecasting ecosystems that combine complementary learning paradigms, heterogeneous environmental observations, and physically informed modeling. This transition marks a broader transformation in environmental AI, shifting from architecture-focused research toward intelligent forecasting systems capable of representing the complex spatiotemporal processes that govern extreme rainfall.

A central conclusion of this review is that forecasting performance depends on factors beyond neural network architecture. Reliable prediction arises from the interaction among high-quality observations, robust data engineering, multimodal information fusion, advanced learning strategies, uncertainty quantification, rigorous verification, and the incorporation of physical knowledge. Rather than identifying a universally superior model, contemporary research increasingly shows that effective forecasting results from integrating complementary methodologies within scientifically consistent and operationally robust frameworks.

Despite these advances, significant scientific and operational challenges persist. Ongoing limitations, including data scarcity, class imbalance, model generalization, benchmarking, uncertainty quantification, computational scalability, and operational deployment, continue to restrict the widespread adoption of AI-based forecasting systems. Addressing these challenges requires methodological innovation, reproducible benchmarking, standardized evaluation protocols, trustworthy AI, interdisciplinary collaboration, and closer integration among atmospheric science, hydrology, and ML. The future of ERP depends on developing adaptive, physically informed, transferable, and uncertainty-aware forecasting systems that remain reliable under increasingly non-stationary climatic conditions. Progress requires building intelligent forecasting ecosystems that integrate data-driven learning with physical understanding and provide transparent, operationally actionable guidance for EWS and climate-risk management. Ensuring these advances are transferable across diverse climatic regions and accessible to both data-rich and data-sparse environments is also essential.

Improving ERP is both a computational challenge and a societal imperative. As climate change intensifies hydro-meteorological extremes, the effectiveness of AI will be measured by its ability to deliver trustworthy, operationally deployable forecasts that enhance disaster preparedness, climate adaptation, resilient infrastructure planning, and sustainable water resource management. Achieving this objective requires sustained collaboration among atmospheric scientists, hydrologists, computer scientists, operational forecasting agencies, emergency managers, and policymakers to ensure that advances in AI lead to measurable reductions in disaster risk and enhanced societal resilience worldwide.
